# Pain-free oral delivery of biologic drugs using intestinal peristalsis–actuated microneedle robots

**DOI:** 10.1126/sciadv.adj7067

**Published:** 2024-01-05

**Authors:** Xize Gao, Jiacong Li, Jing Li, Mingjun Zhang, Jing Xu

**Affiliations:** ^1^ Department of Biomedical Engineering, School of Medicine, Tsinghua University, Beijing 100084, China.; ^2^ Department of Mechanical Engineering, Tsinghua University, Beijing 100084, China.

## Abstract

Biologic drugs hold immense promise for medical treatments, but their oral delivery remains a daunting challenge due to the harsh digestive environment and restricted gastrointestinal absorption. Here, inspired by the porcupinefish’s ability to inflate itself and deploy its spines for defense, we proposed an intestinal microneedle robot designed to absorb intestinal fluids for rapid inflation and inject drug-loaded microneedles into the insensate intestinal wall for drug delivery. Upon reaching the equilibrium volume, the microneedle robot leverages rhythmic peristaltic contraction for mucosa penetration. The robot’s barbed microneedles can then detach from its body during peristaltic relaxation and retain in the mucosa for drug releasing. Extensive in vivo experiments involving 14 minipigs confirmed the effectiveness of the intestinal peristalsis for microrobot actuation and demonstrated comparable insulin delivery efficacy to subcutaneous injection. The ingestible peristalsis-actuated microneedle robots may transform the oral administration of biologic drugs that primary relies on parenteral injection currently.

## Teaser

Bioinspired microneedle robot uses intestinal peristalsis for oral delivery of biologic drugs to replace parenteral injection.

## INTRODUCTION

Throughout the history of human pharmacological therapy, oral delivery has always been a preferred drug administration route due to its low expertise requirements and high patient compliance (*1*, *2*). However, most biologic drugs (peptides, proteins, nucleic acids, and antibodies) are readily deactivated once exposed to the harsh gastrointestinal biochemical microenvironment and are unable to transport across the mucus or cellular layers because of the conservative absorption selectivity of gastrointestinal tract (*3*–*5*). As a result, the oral availability of biologic drugs is limited (about 1%) and must be parenterally injected (*6*–*8*), which may inevitably cause pains and skin infections and often results in poor patient compliance, especially among individuals with chronic diseases that have to perform therapeutic injection frequently (*9*, *10*). Although current oral delivery technologies such as mucoadhesive patches, resident hydrogels, microdevices, and particle-based platforms have been proposed in the open literature to improve topical concentration and prolong release period of biologic drugs, the physiological barriers still exist and clinical efficacy remains to be validated (*11*–*16*). Methods to overcome the oral administration dilemma of biologic drugs and enhance patients’ compliance remain a grand challenge.

Recently, gastrointestinal microneedle devices, which can inject drugs directly into the insensate gastrointestinal wall with minimal perception of pain and discomfort, are being actively pursued as a promising alternative for oral drug delivery (*17*–*21*). Triggered by precompressed springs or balloons ready for expansion, elaborate microneedle devices are able to pierce the drug-loaded microneedles into the capillary networks of gastrointestinal wall to facilitate drug absorption (*17*–*20*). Incorporated with magneto-responsive particles, microneedle devices can be actuated by an external magnetic field (*21*). Through bypassing the biochemical and mucosa barriers, microneedle injections may achieve fast pharmacokinetics (at peak levels at 30 min after deployment) (*17*–*19*) and improved oral bioavailability (>10%) comparable to those of parenteral injections (*17*–*20*). These devices accommodate dosage forms of compressed drug solids or highly concentrated drug liquids, allowing for substantial drug loading from submilligram to multimilligram doses (*17*–*19*). The physical penetration modes also reflect their universal suitability to deliver a broad range of biologic agents (*19*, *20*). However, the spring- or balloon-actuated one-shot mode discussed above in the literature may not guarantee reliability of the microneedle penetration. In contrast, the field-controlled drive mode may still require professional operations. Moreover, using metallic springs, nondegradable elastomers, or magnetic particles as actuation modules often raises concerns from patients and medical professionals. Distinctly, these concerns provide an opportunity to design and engineer versatile ingestible robotic devices for oral drug delivery with patient compliance.

In addition, instead of prestoring energy or inputting external energy for actuation, gastrointestinal motility during normal digestion may provide intrinsic and continuous contraction forces to potentially initiate the microneedle injection (*22*–*25*). Intestinal peristalsis is the coordinated involuntary intestinal contraction and relaxation during digestion for food mixing and propelling (*24*, *26*). The human intestine peristalsis was reported to radially contract about 10 times per minute and compress about 0.18 to 0.5 N/cm (*25*, *27*), comparable to the level of forces needed for microneedle injection and positively correlated to the chyme size (*24*, *25*, *28*). Through the volumetric changes, the peristaltic contraction force generated has the potential to push the microneedles to overcome the resistance for piercing the luminal wall.

**Fig. 1.**
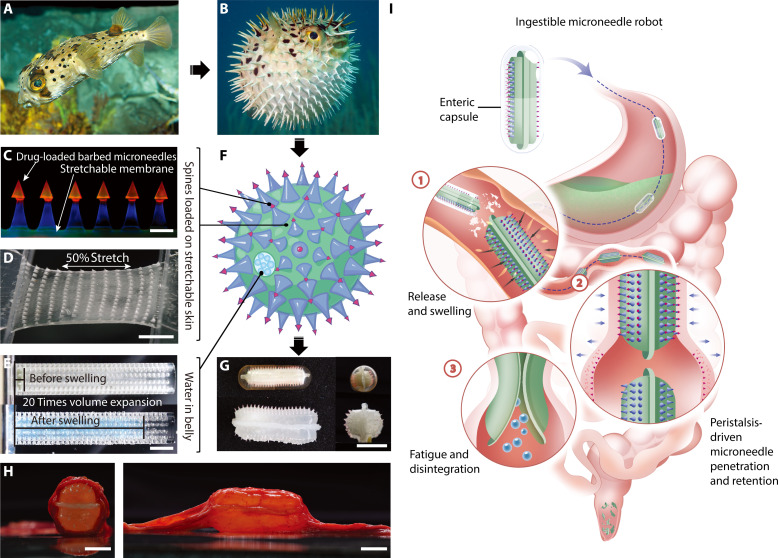
Intestinal peristalsis–actuated porcupinefish-inspired microneedle robots for oral delivery of biologic drugs. (**A** and **B**) Photographs of a porcupine fish (*Diodon holocanthus*) before (A) and after (B) inflation. The fish inflates its own body and erects spines to defend itself when threatened. (A) is reprinted with permission from John White. Copyright 2005 John White. (B) is reprinted from Prescriber, 31: 28–32. Copyright 2020, with permission from Wiley Interface Ltd. (**C**) A fluorescent image showing the stretchable membrane and barb-equipped microneedles. Scale bar, 1 mm. (**D**) Photograph showing the stretched microneedle patch. Scale bar, 5 mm. (**E**) Photograph of the superabsorbent hydrogel before (top) and after swelling (bottom). Scale bar, 15 mm. (**F**) Schematic of the bioinspired microneedle robots containing drug-loaded microneedles, stretchable membrane, and superabsorbent hydrogel, which mimics the spines, skin, and water in belly of the porcupinefish, respectively. (**G**) Photographs of the microneedle robot before and after swelling. Scale bar, 10 mm. (**H**) Photographs of a swollen microneedle robot in ex vivo minipig intestine. Scale bar, 10 mm. (**I**) Schematic of the delivery route of the microneedle robots through a digestive tract. The microneedle robot swells and uses inherent intestinal peristalsis to achieve microneedle penetration, mucosa retention, and drug release. Upon prolonged peristaltic contraction, the microneedle robot reaches its fatigue limit, disintegrates into pieces, and is excreted through the gastrointestinal tracts.

In nature, porcupinefish (*Diodon holocanthus*) expands rapidly by imbibing water when threatened (*29*). During this process, stiff and tapered spines across the fish’s body erect, increase its body size, and stab enemies when being swallowed (Fig. 1, A and B) (*29*, *30*). Conceivably, a porcupinefish-inspired expandable microneedle device, which could rapidly inflate in volume and stick out drug-loaded microneedles toward the intestinal wall, may leverage the peristaltic force during the peristalsis to penetrate the intestine mucus layer and deliver biologic drugs. Meanwhile, certain stinging creatures grow protruding barbs on their spines to enhance adhesion to tissues after penetration (*31*–*33*). This mechanism may inspire a barbed microneedle structure that can be separated from the microneedle device and retained in tissues for drug delivery as well as sustained release.

In this research, an intestinal peristalsis–actuated microneedle robot inspired by the porcupinefish was proposed to assist the arduous oral administration of biologic drugs (Fig. 1, A and B). To break the drug absorption barriers, we designed the microneedle robot to swell into a large spiny vesicle in the intestinal fluids and inject the microneedles into the mucosa while being squeezed during the peristaltic contraction (Fig. 1F). The microneedles were equipped with drug-loaded barbs, and the barbs can be separated from the microrobotic body and strand into the mucosa during the peristaltic relaxation for drug release. Different from the reported spring/balloon-launched or field-controlled actuation mechanisms (*17*–*21*), the proposed microrobotic device functions through both mass transport (imbibing intestinal fluids) and inherent intestinal peristalsis, requiring no prestored energy or real-time external energy input to deliver the drugs into the luminal wall. The microneedle robot (Fig. 1F) mainly contains three parts: (i) drug-loaded barbed microneedles to penetrate mucosa and retain for sustained drug release (spines, Fig. 1C), (ii) a stretchable basal membrane to load microneedles and resist continuous peristaltic contraction (skin, Fig. 1D), and (iii) superabsorbent hydrogel particles to provide swelling stress to balance the membrane tension and puff up the device (water in belly; Fig. 1E). In dry conditions, the single microneedle robot can be encapsulated into a commercial 00# enteric capsule, which keeps them intact through acidic gastric juices and releases them in the gut (Fig. 1G). Upon being exposed to the intestinal fluids, the microneedle robot quickly swells to equilibrium with a desired size [comparable to the Food and Drug Administration (FDA)–approved PillCam and smaller than the diameter of gut lumen], and the stretched membrane is strained to support the spines erecting toward the luminal wall (movie S1 and Fig. 1, G and H). Because of the volumetric effect, rhythmic intestinal peristaltic contraction can push the microneedles into the mucosa, while the relaxation process separates the barb-equipped and drug-loaded tips from the microneedle robot and locks the drug-loaded barbs into the intestinal wall (Fig. 1I). The microneedles can then swell and release the drug into the intestinal vascular and lymph capillary networks. Upon prolonged peristaltic contraction, the microneedle robot reaches its limit for fatigue, disintegrates into pieces, and is excreted through the gastrointestinal tracts. During our repeated in vivo animal tests, no intestinal obstruction was observed because of the optimized equilibrium size, compressibility, and ability of disintegration under continuous peristaltic contraction. Histological analysis of the intestinal tissue after the deployment of the microneedle robot demonstrated fast recovery and limited inflammation. Using insulin as a model drug, we validated fast hypoglycemic effect within 60 min and improved oral bioavailability up to 23.6% during 4-hour observation on minipigs in vivo, considerably higher than the approximately 1% bioavailability reported in current oral administration of biologic drugs (*6*–*8*). This peristalsis-actuated robot may serve as a versatile platform for biologics oral administration that currently relies on injection routes.

## RESULTS

### Construction and characterization of the microneedle robot

**Fig. 2.**
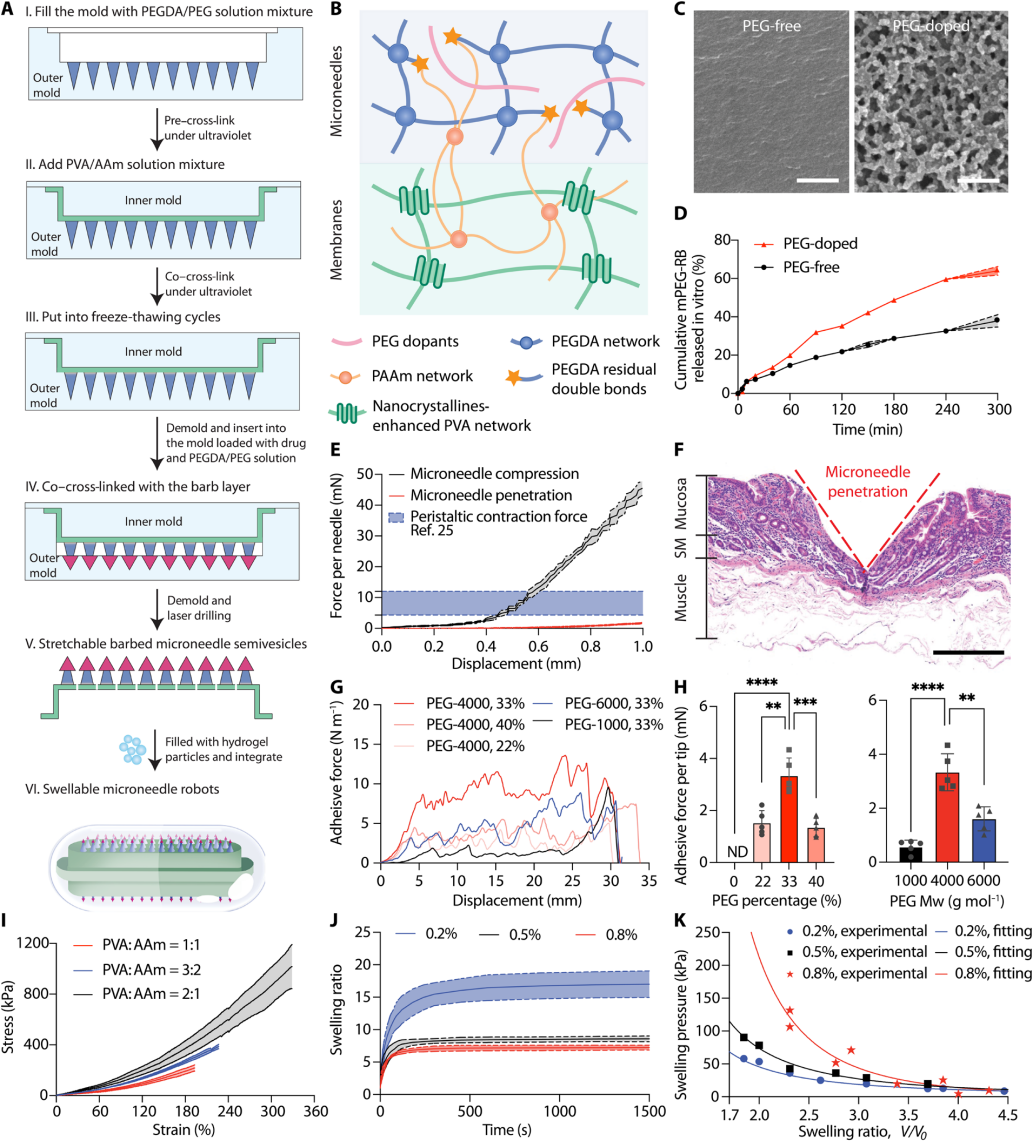
Preparation and characterization of the microneedle robots. (**A**) Workflow to fabricate the swellable microneedle robots. (**B**) Schematic of the rigid-flexible coupling between the microneedles and membrane through covalent cross-linking and physical entanglement. PAAm forms double network with polyvinyl alcohol (PVA). PEG doping leads to lower cross-linking degree inside the microneedle network and more PEGDA residual double bonds left on the microneedle surface as PAAm clicking sites, thereby improving drug release and enhancing the interfacial strength. (**C**) Representative cryo-SEM images of the PEGDA network with (right) and without (left) PEG doping. Scale bar, 2.5 μm. (**D**) In vitro accumulated release of rhodamine-labeled mPEG-5000 in simulated intestinal fluids at 37°C. *n* = 3 technical replicates. (**E**) Compressive (black) and penetration (red) force-displacement curves of the microneedles confirming the robustness of the microneedles and the force required to penetrate into a freshly dissected ex vivo minipig intestine. *n* = 3 technical replicates. The purple band represents the potential range of peristaltic forces acting on the microneedles calculated based on the data from (*25*). (**F**) Representative histological image of the penetrated ex vivo small intestine tissue. Scale bar, 0.5 mm. Red dotted line denoted locations of microneedle penetration. (**G**) Representative force-displacement curve showing the adhesive force between the microneedle and membrane with various PEG molecular weights and doping percentages tested by 90° peeling experiments. *n* = 5 technical replicates. (**H**) Calculated adhesive force per microneedle at the needle-membrane interface, ND, nondetectable, two-tailed Student’s *t* test; PEG percentage, ***P = 0.0004, **P = 0.0012; PEG molecular weight, ****P < 0.0001, **P = 0.0019. (**I**) Tensile stress-strain curves of the membranes with various PVA/AAm ratios. *n* = 3 technical replicates. (**J**) Swelling ratio versus time of hydrogel particles with different cross-linking densities. *n* = 3 technical replicates. (**K**) Swelling pressure versus swelling ratio of hydrogel particles with different cross-linking densities.

To leverage the peristaltic contraction force for the microneedle robot actuation and microneedle penetration, the microneedle robots should be expandable to acquire sufficient peristaltic pressure and stick out their microneedles toward the intestinal wall. A schematic of the construction process of the swellable microneedle robots is illustrated in Fig. 2A. The microneedles of the microrobots were fabricated from a polymeric mixture of polyethylene glycol diacrylate (PEGDA) and polyethylene glycol (PEG) by template-assisted photocrosslinking (Fig. 2AI). PEGDA was chosen as the matrix because of its recognized biocompatibility and desired mechanical strength (*34*). Usually, compact PEGDA network formed through fast cross-linking has small mesh size for drug release (*34*, *35*) (Fig. 2C, left) and leaves limited double bonds for further microneedle-membrane connection (fig. S4). To overcome the above concerns, we added PEG as a diluent to hinder the cross-linking of PEGDA. Cryo–scanning electron microscopy (cryo-SEM) microscopic images confirmed that the PEG-doped PEGDA network had a higher porosity and larger mesh size than the PEG-free network (Fig. 2C). Embedded with the fluorescent dyes, we confirmed up to 50% drug release within 3 hours when doped with 33–wt % polyethylene glycol, molecular weight 4000 (PEG-4000) (Fig. 2D), which attributes to the looser network and the dissolution of hydrophilic PEG. We have also considered using other biocompatible hydrophilic polymers such as polyvinyl alcohol (PVA) and polyvinyl pyrrolidone as the diluent to regulate the drug release rate. However, these materials experienced rapid phase separation after mixing with PEGDA, which means that it became harder to adjust the cross-linking density in the hydrogel network and the promoted drug release was mainly due to the rapid dissolution of the hydrophilic polymers (fig. S5). As determined by compression tests (Fig. 2E), the mechanical strength of the microneedles before buckling was at least 30 mN per microneedle, and the penetration force of the ex vivo porcine intestine was 2.5 mN per microneedle. The mechanical strength of microneedles decreased as PEG percentage and PEG molecular weight increase (fig. S6), which indicated a decreased cross-linking degree as also evidenced by the greater swelling degree (fig. S6). Histology of the punctured tissue showed that the microneedles successfully penetrated the mucosa of the intestinal wall (Fig. 2F). Considering the potential intestine perforation risk, we performed multiple perforation tests and found that a minimum force of 50 mN and a displacement of at least 4 mm toward the tissue were required to cause tissue perforation (fig. S7). These results demonstrated the robustness of the PEGDA/PEG microneedles and the safe tissue penetration without perforation risk under peristaltic contraction pressure.

To regulate the connections between the rigid microneedles and the flexible membranes, the membrane mixture solution containing PVA and acrylamide (AAm) monomers were transferred into the mold and co–cross-linked with the microneedles under ultraviolet light, during which PAAm formed double networks with PVA and linked with PEGDA by covalent bonds (Fig. 2, AII and B). As such, the density of covalent bonds formed at the interface influences the connection force between the two phases. By adjusting the PEG percentage or the PEG molecular weight, more double bonds of PEGDA can be formed on the microneedle surface as PAAm clicking sites (fig. S4), so that they can be used to enhance the adhesion of the interface (Fig. 2G and fig. S4). Specifically, when doping PEGDA with the same amount of PEG-4000, the microneedles could bear 3.3 mN of peeling force per microneedle before detachment, which is regarded as a tight connection at the interface (Fig. 2H). In addition, the ratio of AAm to PVA modulated the connection force by adjusting the number of covalent bonds between PAAm and PEGDA (fig. S8). Through freeze-thawing cycles, PVA nanocrystallines formed as network junctions (*36*) and microneedle-loaded PVA/PAAm membranes with fracture strength up to 1.3 MPa were obtained (Figs. 2AIII, 1D, and 2I). By changing the ratio of AAm to PVA, we obtained stretchable membranes with adjustable Young’s modulus (Fig. 2I). To obtain the barbed microneedles, the microneedle patch was inserted into another mold and co–cross-linked with the insulin-preloaded PEGDA/PEG solution (Fig. 2A, IV and V). The photocrosslinking process resulted in a high insulin encapsulation efficiency close to 100% with a loading capacity of up to 6% for the microneedles. This approach led to the loading of 0.5 mg of insulin on 126 tetragonal barbs (height, 800 μm; underside length, 500 μm) of each microneedle patch and 1.0 mg of insulin on a single microneedle robot. Percentage of potentially cytotoxic unreacted monomers were analyzed by ultraperformance liquid chromatography (UPLC). The results confirmed that the amount of residual monomers (< 0.2%) remained within the FDA safety limits (fig. S9) (*37*).

To swell the microneedle robot, superabsorbent hydrogel particles synthesized from high-viscosity sodium carboxymethyl cellulose (SCMC) were stuffed into the pocket glued by two stretchable microneedle semi-vesicles (Fig. 2AVI). Because of the abundance of hydroxyl groups and charges on the molecular chain, SCMC especially those with high viscosity can absorb and retain fluids largely (*38*). Cross-linked by citric acid, SCMC transformed into superabsorbent hydrogels with adjustable swelling volume ratios, which increased with the decreasing of cross-linking density and reached up to 20 times at 0.2% citric acid content (Fig. 2J). In addition, hydrogel particles of different cross-linking densities reached swelling equilibrium in about 10 min (Fig. 2J). This indicates that these hydrogel particles have a high swelling speed. On the basis of the ideal elastomeric gel model, the swelling pressure of hydrogel decreases with the swelling process until it equilibrates with the elastic restoring stress (*39*). Figure 2K shows the swelling pressure of SCMC hydrogel as a function of swelling ratio. At low swelling ratio, the swelling pressure of the hydrogel reaches hundreds of kilopascals, which is sufficient to deform the membrane and swell the robot (fig. S10). Last, the integrated microneedle robots were filled into a commercial 00# capsule coated with enteric membrane (Eudragit L100) to avoid early swelling in the stomach (fig. S11).

Notably, materials used to fabricate the microneedle robot have been extensively used in medicine (*36*, *40*, *41*) and have shown promising biocompatibility in the in vitro biocompatibility tests (fig. S12). Following a 24-hour coculture with the material extracts, the relative viability of mouse embryonic fibroblasts exceeded 80%, and no obvious cytotoxicity was observed (fig. S12). The materials have been also reported to exhibit biodegradability, facilitated either by endogenous enzymes (*41*) or gastrointestinal microorganisms (*34*, *42*–*44*). In vitro degradation tests confirmed the materials’ capability to degrade in simulated intestinal fluids (SIFs), which is crucial for safe metabolism and excretion (fig. S13).

### Swellable microneedle robots using peristaltic contraction to promote mucosa penetration

**Fig. 3.**
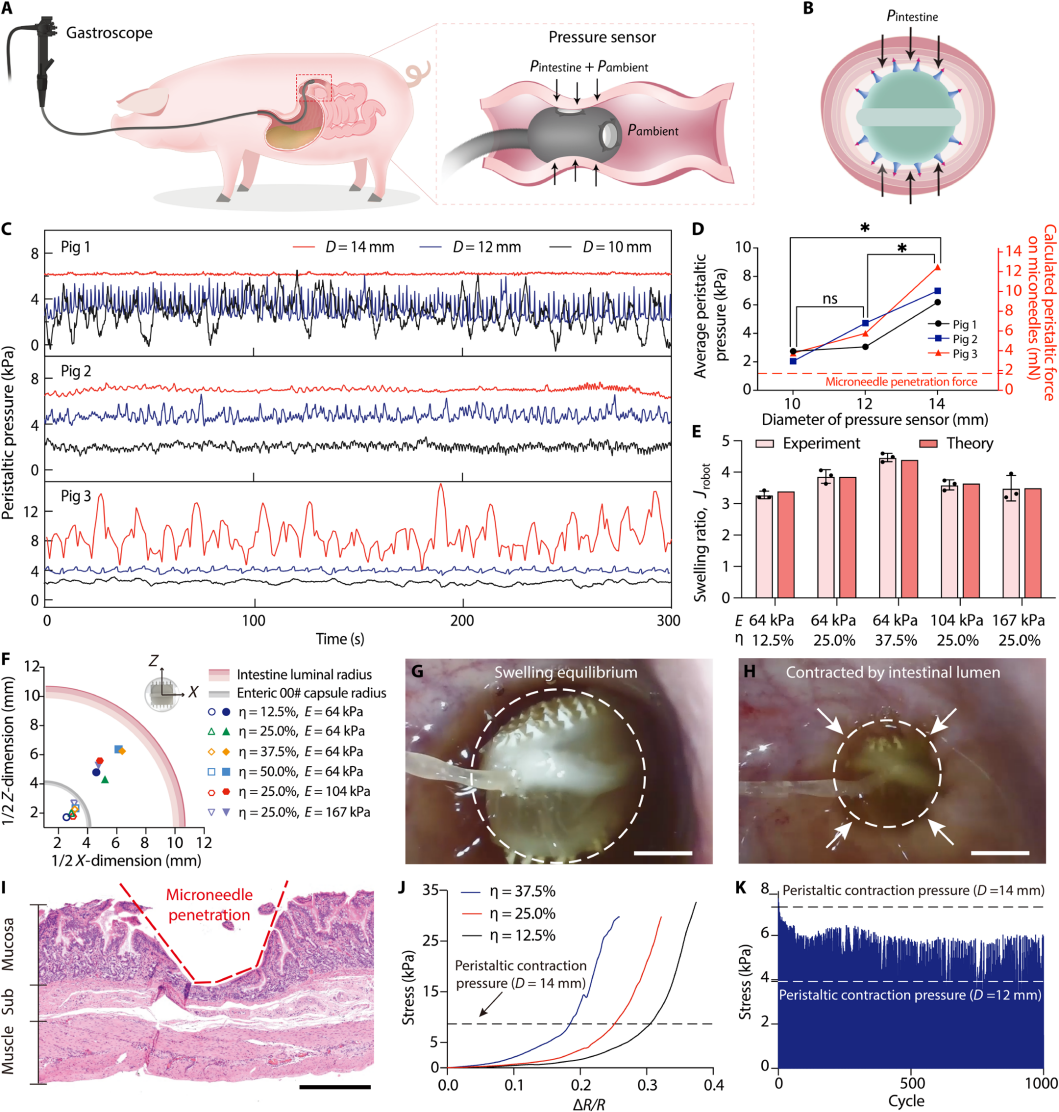
Microneedle penetration through peristaltic contraction. (**A**) Schematic of in vivo measurement of intestinal peristaltic contraction force of the minipigs using a pressure sensor attached to a gastroscope. (**B**) Schematic of the peristaltic pressure (*P*_intestine_) acting on the microneedles. (**C**) Representative excerpted 5-min peristaltic pressure profile of the three minipigs measured by pressure sensors with diameters of 10, 12, and 14 mm. *n* = 3 biological replicates. (**D**) Calculated peristaltic force acting on the microneedles of the microrobot with diameters of 10, 12, and 14 mm. Two-tailed Student’s *t* test, 12 versus 14, *P = 0.0405; 10 versus 14, *P = 0.0236; 10 versus 12; ns, nonsignificant. (**E**) Comparison of the experimental and theoretical expansion volume values of the microneedle robots with various parameter values. *n* = 3 technical replicates. (**F**) Dimensional change of the microneedle robots of various hydrogel loading ratios and membrane modulus before and after the swelling. (**G** and **H**) Gastroscopic images showing a microneedle robot at the swelling equilibrium state (G) contracted by the intestinal lumen (H). Scale bar, 5 mm. (**I**) Histology staining sections showing efficiency of the in vivo penetration. Scale bar, 0.5 mm. Red dotted line denotes locations of microneedle penetration. (**J**) Representative compressive curves of the microneedle robots at the equilibrated state of different hydrogel loading ratios and membrane modulus. *n* = 3 technical replicates. (**K**) Compressive tests of 1000 compression cycles of the equilibrated microneedle robots under peristaltic pressure.

Given the strength of intestinal peristalsis positively correlates to the chyme size (*24*–*26*), the volume of the robot at swelling equilibrium state may determine the peristaltic contraction force acting on the robot, thus affecting the penetration effect of the drug-loaded microneedles. To optimize the proper size of the microneedle robot, Bama minipigs, whose gastrointestinal tracts are anatomically and physiologically similar to that of humans (*45*), were chosen as the in vivo animal models for intestine manometry and microneedle penetration evaluation. As illustrated in Fig. 3A and fig. S14, a series of custom-designed pressure sensors of various diameters were delivered into the small intestine by a gastroscope to measure the peristaltic pressure in vivo in real-time. To eliminate the ambient pressure, we installed the pressure measurement units at both the axial and radial ends of the capsule-shaped pressure sensor, and the pressure difference was obtained as the intestinal peristaltic pressure (*P*_intestine_) (Fig. 3A and fig. S14). Figure 3C shows the excerpted 5 min of peristaltic pressure profile of three minipigs measured by pressure sensors of 10, 12, and 14 mm in diameter. For each minipig, sustained peristaltic pressure with a primary frequency of about 10 cpm (fig. S15) was observed, and an unpaired *t* test showed a significant increase of average pressure when tested with larger sensors (Fig. 3D). Because the peristaltic pressure derives from the impulse of the intestinal motility, it is assumed that the same contraction force is applied on the microneedle robot with the same diameter as the sensor. The contraction force exerted on each microneedle (*F*_intestine_) could be converted from the peristaltic pressure (Fig. 3B; see note S1 for details). The calculation results suggested that when the diameter of the microneedle robot reaches 14 mm, *F*_intestine_ may reach 10.5 ± 1.3 mN on a single microneedle, which is four times higher than the penetration force needed as reported in the open literature (*18*) (Fig. 3D). These data showed that in the minipig cohort, increasing the radial dimension of the robotic device can increase the peristaltic pressure acting onto it, thereby providing desired driving force for the microneedles to penetrate into the intestinal wall.

For the swellable microneedle robot that contains inner superabsorbent hydrogels and outer stretchable membranes, the internal swelling pressure (*P*_swelling_) is balanced with the membrane tension (σ_membrane_) (*46*). During swelling, the pressure can be predicted by ideal elastomeric gel model (*39*, *47*), and the membrane tension can be converted from the tensile stress-strain curve of the membranes (Fig. 2I; see note S2 for details). As a result, the swelling ratio of the microneedle robot (*J*_robot_) can be predicted, which is mainly determined by the hydrogel filling ratio (η) and the membrane modulus (*E*) (see note S2 for details). To verify the predicted results experimentally, we compared *J*_robot_ of the microneedle robots with various values of η and *E* with the theoretical values. As shown in Fig. 3E, the swelling ratio of the microneedle robots is consistent with the theoretical values. Figure 3F shows comparison of the radial dimensions of the microneedle robots with various parameter values under dry and swollen conditions. Under the dry conditions, the microneedle robots can be encapsulated in a commercially available 00# enteric capsule (diameter = 8 mm). In a fluidic environment, the robot swells to the equilibrium state, with an increasing diameter from 10 to 14 mm as η increases, which will generate enough intestinal contraction pressure to push the microneedles into the intestinal wall. Gastroscopic movie S2 shows a swollen microneedle robot being squeezed by the peristaltic intestinal wall. When the intestine relaxed, the microneedle robot appeared to be much smaller than the intestine diameter (Fig. 3G). When the intestine contracted, the intestinal wall squeezed the microneedle robot tightly (Fig. 3H), and the tissue sliced from a minipig after euthanasia verified the microneedle penetration actuated by intestinal peristalsis (Fig. 3I). Further histological analysis indicated complete tissue recovery and limited inflammation at the penetration sites 1 day and 1 week after administration as shown in fig. S16. To avoid potential obstructions, we designed the radius of the microneedle robot at the equilibrium state to be less than the diameter of the intestinal lumen.

To quantitatively characterize the compression resistance of the microneedle robot against continuous contraction of the intestinal peristalsis, we measured the compressive force-strain curves of the robot under various η and *E* values. The experimental data showed that all the microneedle robots could endure up to 30 kPa of circular compressional pressure before rupture, which is much larger than the peristaltic pressure of the small intestine (Fig. 3J and fig. S17). In addition, when a 25% cyclic strain was applied to the microneedle robot, the compressive stress stayed stable at about 6 kPa (comparable to the peristaltic pressure) over 1000 cycles, confirming the robustness of the robot under continuous physiological compression (Fig. 3K and fig. S17). To balance the swelling ratio and compression resistance, we used the microneedle robots wrapped by a membrane with a modulus of 64 kPa and loaded with 25% hydrogel particles for further study.

### Swellable microneedle robots using peristaltic relaxation and barbed structures to promote mucosa retention

**Fig. 4.**
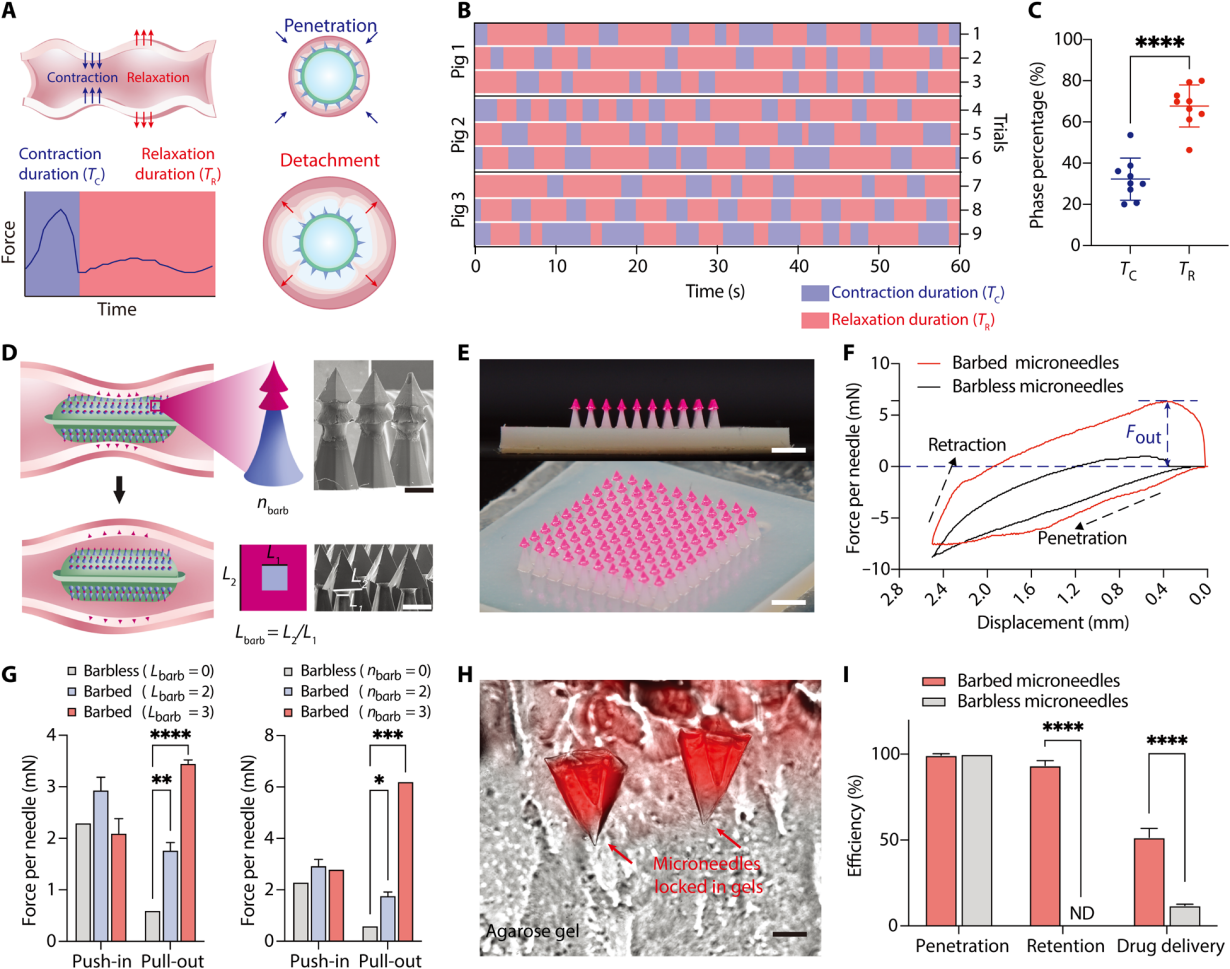
Microneedle retention through peristaltic contraction and relaxation. (**A**) Schematic of peristaltic contraction and relaxation, and the interactions between luminal wall and the microneedle robot during the contraction duration (*T*_C_) and relaxation duration (*T*_R_). (**B**) Analysis of the alternations of contraction and relaxation phases of intestinal peristalsis on three minipigs. (**C**) Statistical comparisons between the durations of contraction and relaxation phases of intestinal peristalsis. *n* = 3 technical replicates each for three biological replicates totaling *n* = 9. Two-tailed Student’s *t* test, ****P < 0.0001. (**D**) Schematic of the barbed microneedle retention through peristaltic contraction and relaxation (left). The barbed microneedles are constructed with different layers (*n*_barb_) and side length ratios (*L*_barb_) to increase the retraction force for microneedle separation and tissue retention (middle). SEM images of the barbed microneedles (right). Scale bar, 400 μm. (**E**) Photographs of the barbed microneedle patches. Scale bars, 2 mm (top) and 1 mm (bottom). (**F**) Representative force-displacement curves of the barbless microneedles and barbed microneedles during the penetration and retraction cycles. *F*_out_ is used to evaluate the retraction force. (**G**) The retraction force of the barbed microneedles with various side length ratio (left) and barb layer numbers (right). Mean ± SD, *n* = 3 technical replicates. Two-way ANOVA test; *P = 0.0247, **P = 0.0067, ***P = 0.0001, ****P < 0.0001. (**H**) Merged microscopic images show the retention of barbs in the agarose gel. Scale bar, 200 μm. (**I**) Comparisons of penetration, retention, and drug delivery efficiency of the barbless and barbed microneedles after penetration-retraction cycles. Mean ± SD, *n* = 3 technical replicates. Two-way ANOVA test, ****P < 0.0001.

The rhythmical nature of intestinal peristalsis implies that it involves not only intense contractions but also a periodic relaxation (*22*) (Fig. 4A). Figure 4B shows the contraction-relaxation alternations in 60 s of three minipigs from the peristaltic pressure profiles (fig. S18). The data confirmed that during the peristalsis, the contraction and relaxation phases continuously alternated, and two-way analysis of variance (ANOVA) test confirmed that the relaxation phase occupied a longer portion of the overall period than the contraction phase for all the minipigs tested (fig. S19), statistically accounting for about 67.7% of the peristaltic cycle (Fig. 4C). However, the prolonged relaxation periods may cause the detachment of the drug-loaded microneedles from the mucosa and expose them to the harsh luminal environment, ultimately reducing the efficiency of the drug delivery (Fig. 4A).

Inspired by the underlying principles of the hard-to-remove barbed spines of certain stinging creatures (*31*–*33*), we constructed a pine-inspired barbed structure for the microneedles to enhance the mechanical interlocking of the microneedle with the fibrous tissues, so that the barbed microneedles are hard to withdraw from the mucosa and readily to detach from the microneedle robot during the peristaltic relaxation (Fig. 4D). The pine-inspired barbed microneedles were constructed by a multiple axial photocuring process (Fig. 2A and 4E), during which the microneedle retraction force (*F*_out_) could be modulated by simply changing the number of barb layers (*n*_barb_) and the side length ratios between two layers (*L*_barb_) (Fig. 4D). At the same time, the connection force between two layers also changes with their overlapping height. To verify feasibility to use the barbed structures for tissue retention, we first tested the retraction force of the barbed tips on ex vivo porcine small intestines (Fig. 4F). Compared with the bare microneedles, the barbed microneedles need up to 6.2 mN per microneedle to retract themselves from the tissues, and the retraction force increased with the increase of the *n*_barb_ and *L*_barb_ values (Fig. 4G), which are attributed to the increasing interlocking effects between the barbed structure and the fibrous tissue (fig. S20).

When the retraction force of the barbed layers is greater than their connection force with the first layer, the barbed tips can be easily separated from the microneedle robot and trapped inside the tissue during the peristaltic relaxation process (Fig. 4D and fig. S20). After 20 times of peristalsis-mimicked thrust-pull cycles, we observed that up to 93.5 ± 2.8% of the microneedles were separated from the robot and were retained in the tissue (movie S3 and Fig. 4, H and I), which will result in increased delivery of drugs into the tissue than the nonretained barbless microneedles. Loading the microneedles with fluorescent dyes, we further investigated the drug delivery efficacy of the barbed microneedles. In the barbed group, most microneedles were trapped in and released up to 51.5 ± 5.3% drugs into the tissues after 1-hour penetration-retraction cycles, while the barbless microneedles could only release drugs into the intestine tissues during penetration, leading to a drug accumulation of only 11.9 ± 0.7% (Fig. 4I and fig. S20). These experiments confirmed the importance of using the barbed structures to achieve tissue retention and sustained drug release through peristaltic relaxation.

#### In vivo drug delivery efficacy and safety

**Fig. 5.**
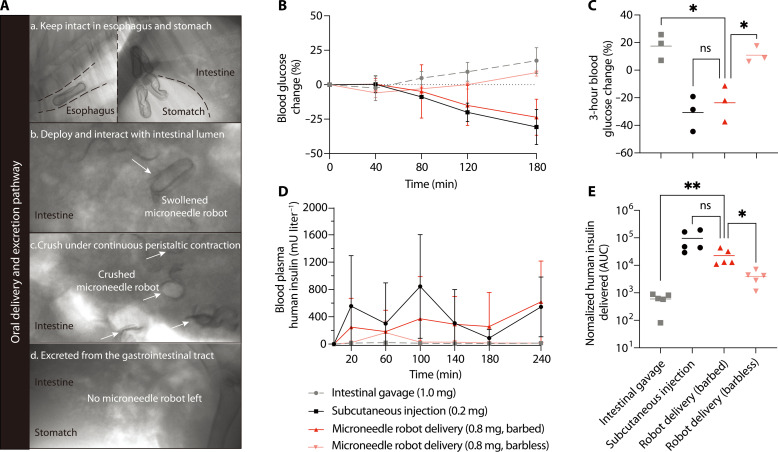
In vivo drug delivery in minipigs. (**A**) X-ray images showing the oral delivery and excretion routes in vivo. The capsule-coated microneedle robots keep intact in the esophagus and stomach (a) and then pass through the pylorus and into the intestinal lumen (b). Once being exposed to the gut fluids, the microneedle robots begin to swell and interacted with the intestinal lumen (c). Last, the microneedle robots are contracted to crush under continuous peristaltic contraction and excreted from the gastrointestinal tracts (d). (**B** and **D**) Blood glucose (mean ± SD, *n* = 3 biological replicates) and plasma human insulin levels (mean ± SD, *n* = 5 biological replicates) tested in minipig after endoscopy-aided intestinal gavage (1.0 mg), subcutaneously injection (0.2 mg), and delivery via robotic capsules with (0.8 mg, barbed) or without (0.8 mg, barbless) barbed microneedles. (**C**) Blood glucose changes after delivered by various delivery methods for 3 hours, two-tailed Student’s *t* test; intestinal gavage, *P = 0.0117; robot, *P = 0.0142. (**E**) Normalized human insulin delivered into the blood plasma within 4 hours calculated by the area under the plasma concentration-time curve (AUC) divided by dose, two-tailed Student’s *t* test; *P = 0.0226, **P = 0.0099.

To visualize the oral delivery and excretion routes of the microneedle robot, we first trans-orally administered five microneedle robots loaded with x-ray contrast (fig. S21) into a minipig, and their gastrointestinal distributions were observed under fluoroscopy (Fig. 5A and fig. S22). Because the robot is small enough to fit into a commercial 00# enteric capsule, it kept intact in esophagus and stomach and passed into the intestinal lumen after gastric emptying (Fig. 5A, a and b). Through the experiments, we observed the size of the microneedle robot affects the efficacy of gastrointestinal transit. Because of the slow gastric emptying times over 24 hours and the narrow pylorus in minipig models (*48*), the prepared 30-mm-long microneedle robots did not appear in the intestine until the fifth day, and the long pylorus-passing time caused four of the five microneedle robots to be crushed into pieces by intense gastric contractions (fig. S22A). To enable more microneedle robots to enter the intestine intact, additional five 15-mm-long (a half-length of the first batch) microneedle robots with the same diameter as the first batch were fabricated and carried out the same delivery experimental procedure. The results demonstrated that the shorter microneedle robots passed through the pylorus 1 day earlier than the longer ones (on day 4), and four of these five entered the intestine completely (fig. S22B). Once inside the intestine, the microneedle robots began to swell and interact with the intestine (Fig. 5Ab). Last, the microneedle robots were squeezed to be crushed under continuous peristaltic contraction (Fig. 5Ac) and completely excreted from the gastrointestinal tracts on day 6 and day 7, respectively for the shorter and the longer microneedle robots (Fig. 5Ad and fig. S22). These data indicated that the microneedle robot could pass through the gastrointestinal tracts, and its size influenced the transit time as well as the ability to enter the intestine intact to perform functions. Considering the faster gastric emptying time and the larger pylorus of humans than minipigs (*49*, *50*), the microneedle robots may be optimized to traverse the human gastrointestinal tract in less time, making it less likely to be destroyed inside the human stomach, which is an advantage compared to the minipig stomach.

To verify the drug delivery efficacy, we further loaded human recombinant insulin into the microneedle robots, and measured the changes of blood glucose and plasma drug concentration of the minipigs within 4 hours after in vivo deployment. To circumvent the long gastric emptying time and identify the observation starting point, we directly delivered the microneedle robots into the duodenum and continuously observed them using a gastroscope after animal anesthesia (fig. S23 and movie S2). Our robotic delivery method (0.8 mg) was compared with subcutaneous injection (0.2 mg) and intestine gavage (1.0 mg) of insulin solution. During the sampling period, the robotic delivery method achieved a 23.6 ± 10.7% blood glucose decrease over 180 min, comparable to the 30.7 ± 10.4% decrease of subcutaneous injection (*n* = 3). The intestine gavage groups did not show obvious blood glucose decrease but increased slightly after 40 min (Fig. 5, B and C). Consistent with the observation, pharmacokinetic studies show that the robotic delivery method achieved comparable high-dose insulin delivery to subcutaneous injection, and its relative bioavailability reached 23.6% (*n* = 5), 37.7 times that of the intestine gavage group (0.6%, *n* = 5) (Fig. 5, D and E). To demonstrate the contribution of the barbed structure, we also investigate the delivery efficacy of the barb-free microneedle robots. Notably, the hypoglycemic effect or bioavailability of the barbed group was significantly higher than that of the barb-free group (4.1%, *n* = 5), and there was no significant difference between the barb-free group and the intestine gavage group (Fig. 5, C and E), which confirmed the importance of the barbed structure in microneedle design to ensure the long-term drug release in tissues. No intestinal obstruction was observed during delivery and subsequent health monitoring, and no intestinal bleeding or perforation was observed by the microneedle penetration (fig. S16). Because of the high turnover of the mucus and epithelial linings, the epithelium damaged by the microneedles quickly healed (*51*–*53*) (fig. S16). During the week after delivery, we continuously monitored the daily activities of the minipigs and no obvious abnormalities such as intestinal obstruction, altered fecal formation, feeding habit change were found. We attribute this to the compact size, compressibility, and ability of disintegration under continuous peristaltic contraction exhibited by the microneedle robots. These experiments validated the efficacy and safety of the microneedle robots as an oral delivery platform for biological drugs.

## DISCUSSION

In this research, we have developed an unconventional intestinal peristalsis–actuated microneedle robot and demonstrated its feasibility for intestine microinjection as well as oral delivery of biologic drugs in minipigs. Inspired by the inflating and stinging defensive behaviors of the porcupinefish when threatened, the microneedle robot can swell into a volume-enlarged spiny vesicle by absorbing intestinal fluids and leverage the radial peristaltic contraction to push the drug-loaded microneedles into the intestinal wall. The equilibrium volume of the microneedle robot was optimized to have sufficient peristaltic pressure for microneedle penetration and avoid causing intestinal obstruction. The moderate and repeated piercing processes ensure high drug delivery reliability and can effectively reduce the penetration miss and perforation risk compared with the intense one-shot actuation mode (*17*–*21*). The barbed microneedle structure enables easy separation of the drug-loaded microneedles from the robot body after insertion and promotes their retention in the intestinal wall during the peristaltic relaxation. In vivo drug delivery experiment in minipigs demonstrated a distinct hypoglycemic effect and a bioavailability of insulin up to 23.6% (Fig. 5, B to E), which exceeds the bioavailability in typical biologics oral administration (*6*, *7*) and stands high among the enteral robotic pills for insulin delivery (~10%) reported in the open literatures to date (*18*, *21*). A single microneedle robot can accommodate 1.0 mg of insulin, and the loading capacity can be safely doubled (2.0 mg) for human use by increasing both the equilibrium diameter and the number of microneedles in the robot, which may allow the system to meet the clinical dosage requirement (1.2 mg) for a 70-kg human adult (*19*, *54*, *55*). In addition, the proposed microrobotic delivery method may be used for multidrug cocktail delivery and combined with micro-nano delivery systems for controlled release as well as stimuli-responsive release of a wide range of biologic drugs (*55*). These results suggest that the peristalsis-actuated microneedle robot presents a promising approach to oral drug delivery by leveraging intestinal contraction as well as relaxation.

Compared with the existing gastrointestinal microrobotic devices for drug delivery, the peristalsis-actuated microneedle robot leverages intrinsic physiological motility and the volumetric effects of their own expansion to achieve robotic actuation. This peristalsis-driven mode requires no prestored energy or external input during the execution, thereby avoiding the use of nonbiodegradable actuation modules such as metallic springs, rubber elastomers, and magnetic particles (*17*–*21*). It is noteworthy that the materials used to fabricate the microneedle robots are biocompatible and biodegradable, which is of particular value for translational clinics. Moreover, gastrointestinal peristalsis, the rhythmic contraction and relaxation of the muscles in the digestive tract, is widely observed in humans (*25*, *27*). By leveraging the inherent peristaltic motion of the gastrointestinal tract, our proposed microrobotic device can seamlessly integrate with the body’s natural processes, offering a more harmonious and efficient approach to biologic drug delivery. This physiological compatibility further reinforces the feasibility and relevance of our research in the field of human medicine.

Nevertheless, gastric emptying time of the microneedle robot should be considered as one of the key factors influencing the efficacy of oral drug delivery for human patients (*50*). While the microneedle robot has demonstrated notable higher bioavailability than current insulin oral administration, more than 72 hours were required for the microneedle robots to be transited into the small intestine for minipigs (fig. S22). However, the transit time of nondissolving solids for humans (1.2 to 2 hours) has been reported much faster than that of minipigs (>24 hours) because of the more relaxed pyloric closure and upright position (*48*–*50*). Through optimizing the mechanical balance between the stretchable membrane and the absorbent hydrogels, the swellable microneedle robot can have a small initial size and a high expansion rate, which may further shorten the gastric transit time. In addition, other parts of the digestive tract such as the stomach exhibit much more intense peristalsis behaviors (*56*), which may serve as another promising target and arena for peristalsis-actuated robots and circumvent the influence of gastric emptying entirely.

In consideration of universal applicability of the microneedle robot to individuals with intestinal motility differences, our design capitalizes on the typical ranges of peristaltic contraction frequency (~10 Hz) and pressure (0.18 to 0.5 N/cm) exhibited by most populations, except for individuals with severe gastrointestinal motility disorders (*25*, *27*). This approach provides a basis for designing microneedle robots that can cater to the physiological characteristics of most patients. In addition, our data reveal that the available intestinal peristaltic pressure increases with the size of the microneedle robot (Fig. 3, C and D). Despite its notable smaller size (10 to 14 mm) than the lumen diameter (20 mm for minipigs), the microneedle robot can withstand and harness sufficient peristaltic forces to enable mucosa penetration (Fig. 3D). This suggests that even individuals with vulnerable gastrointestinal motility may safely benefit by selecting larger microneedle robots within the intestinal diameter (Fig. 3, D and F). The potential for individualized treatments, factoring in specific intestinal motility patterns, would further optimize the robot’s clinical performance (*57*). Nonetheless, future preclinical and clinical research will play a pivotal role in refining the device, thus enhancing its adaptability across a spectrum of peristaltic patterns. In summary, the proposed peristalsis-actuated microneedle robot presents a promising platform for the oral delivery of biologic drugs to replace injection routes, which has the potential to improve patient outcomes and comfort in a wide range of therapeutic areas.

## MATERIALS AND METHODS

### Experimental design

The objective of this study was to investigate the feasibility and efficacy of an intestinal peristalsis–actuated microneedle robot for the administration of biologic drugs. First, the microneedle robots of different swelling equilibrium sizes were prepared by integrating the drug-loaded barbed microneedles, stretchable membranes, and superabsorbent hydrogels. The swelling performance of the microneedle robots was evaluated in SIFs, and the mucosa penetration and retention performances were evaluated using fresh porcine intestines ex vivo. To validate the feasibility of using intrinsic intestinal peristalsis for the actuation of microneedle robots, the real-time peristaltic pressure was measured in vivo using Bama minipigs by a series of custom-designed pressure sensors and the mucosa penetration efficiency was evaluated by histological assays. The interaction between the microneedle robots and the intestinal lumen, the oral delivery and excretion routes of the microneedle robots were investigated by endoscopy and fluoroscopy. Last, the drug delivery efficacy of the microneedle robots was investigated by the application of human insulin–loaded microneedle robots to 12 randomly housed Bama minipigs. The hypoglycemic effect and bioavailability of the microneedle robots were evaluated within 4 hours after in vivo deployment. Certain dose of insulin was administrated to Bama minipigs via gastroscopy-aided intestinal gavage or subcutaneous injection for comparison. All animal tests were approved by and performed according to the animal care and ethics committee of Tsinghua University and Beijing Medical Service Biotechnology Co Ltd.

### Materials

SCMC with a viscosity about 7600 cps was received from Lihong Fine Chemicals (Chongqing, China). Human recombinant insulin was purchased from Psaitong (Tianjin, China). SIF (pH = 6.8, sterile) and simulated gastric fluid (pH = 2, sterile) were purchased from Boer Biotech (Chongqing, China). PVA with an average molecular weight from 89,000 to 98,000 was purchased from Sigma-Aldrich. Sodium citrate, citric acid, AAm, *N*, *N*′-methylenebis (2-propenamide), Irgacure 2959, 2-hydroxy-2-methylpropiophenone (HMP), PEGDA-600, PEG-400, PEG-800, PEG-1000, PEG-4000, PEG-6000, and rhodamine B–labeled mPEG-5000 (RB-mPEG) were purchased from Aladdin. Cell counting kit-8 (CCK-8) was purchased from Meilunbio. Dulbecco’s modified Eagle’s medium (DMEM) and fetal bovine serum were purchased from Thermo Fisher Scientific. The enteric capsules were purchased from DRcaps (Japan). The deionized water was prepared by a laboratory water purification system with a resistivity above 18.2 megohm cm^−1^. Human insulin enzyme-linked immunosorbent assay (ELISA) kit was purchased from i-presci Scientific.

### Microneedle fabrication

Negative polydimethylsiloxane microneedle molds with varying microneedle heights and spacing were designed using SolidWorks and ordered from Microchip Medical Technology (Taizhou, China). Microneedles with varying heights were prepared and characterized in both ex vivo penetration test and in vivo drug delivery validation. Microneedle patches were prepared by a template-assisted photocrosslinking method. Typically, a mixed aqueous solution of 33–vol % PEGDA, 33–wt % PEG, and 1–vol % HMP were poured into the negative mold, degassed using ultrasonic cleaner, and photocrosslinked under an ultraviolet-light lamp (35 mW/cm^−2^, 320 to 390 nm; Uvitron IntelliRay 600) for 10 s. To obtain barb-equipped microneedles, the patches were demolded, inserted into another mold filled with insulin-loaded microneedle solution, and then solidified under ultraviolet light.

### Hydrogel particle preparation

Superabsorbent hydrogel particles were obtained from high-viscosity SCMC by chemical cross-linking. Typically, 3 g of SCMC powder was slowly dispersed in citric acid solution under vigorous stirring. Then, the gelatinous SCMC/citric acid complex was put in a drying oven and cross-linked. The dried hydrogels were ground into small particles with a diameter around 1 mm for later use.

### Basal membrane preparation

The basal membranes of the microneedle robot were prepared by coupling freeze-thawing and photocrosslinking to construct double network. Typically, a mixed aqueous solution of PVA, AAm, *N*, *N*′-methylenebis (2-propenamide), and photoinitiator Irgacure 2959 at mass concentrations of 10, 10, 0.1, and 0.2% was prepared at 80°C and degassed by sonication. The solution was then cast in a 1-mm-thick customized silicone mold, photocrosslinked under ultraviolet light (35 mW/cm^−2^, 320 to 390 nm) for 20 min and put into freeze-thawing cycles. After 5 cycles of freezing and thawing, stretchable basal membranes were obtained and persevered in a humid environment for further mechanical tests. For the connection between rigid microneedles and flexible basal membranes, the mixed membrane solution was poured into the mold and photocrosslinked with PEGDA in the microholes in an ice bath. Then, the molds were put into freeze-thawing cycles as described above and the stretchable microneedle semi-vesicles were obtained after demolding.

### Robot integration

A nanosecond laser marking machine (HAN’S Laser, UV5X) was used to introduce laser holes into the freshly prepared stretchable membrane or stretchable microneedle semi-vesicle. Then, a certain amount of dehydrated hydrogel particles was stuffed into the pocket sealed by two stretchable microneedle semi-vesicles using a biocompatible cyanoacrylate adhesive (Krazy Glue). The integrated robots were then put into a dry chamber and shrunk down to a small size for enteric capsule loading.

### Mechanical characterization

The mechanical properties of the microneedles, membranes, and the integrated microneedle robots were measured by a customized displacement-force test station equipped with a 25-N forcemeter (Mark-10, M4-5) and an electrically controlled moving stage (Daheng Optics, GCD-301101 M). For microneedle compression test, the microneedle patch was glued to the stainless steel moving stage and positioned facing toward the force. The stage moved at a rate of 0.1 mm s^−1^, and the force versus displacement was measured when the microneedles touched the forcemeter. A camera equipped with a macro lens was used to visualize the compression process. All the mechanical tests were repeated three times for each condition.

For tensile test of the stretchable membranes, they were prepared as described above in a shallow dog-bone–shaped mold and were incubated in SIF at 37°C to simulate the physiological conditions before testing. The stress-strain curves were obtained during tests, and the tensile strength and Young’s modulus of the membranes were determined based on the maximum stress and the curve slope.

The compression resistance of the microneedle robots was obtained by circular compression tests (fig. S17). For this, column-shaped microneedle robots of various parameters were prepared and incubated in SIF before testing. The compressive stress-strain curves and the compressive strength of the microneedle robots were obtained during compression. To simulated the continuous peristalsis in the gut, 1000 compression cycles of a 25% strain and a 6-kPa stress were conducted on the robot.

### Adhesion test

To evaluate the adhesive force between the microneedles and the membranes, PEGDA/PEG-PVA/PAAm bilayer strips with various PEG percentages or molecular weights and AAm percentages were prepared. The adhesive force was characterized by a 90° peeling test (fig. S8) using a customized displacement-force test station equipped with a 2-N forcemeter (Weidu Gage, SH-2). Force-displacement curves were conducted with at a rate of 1 mm s^−1^. The adhesive force was determined by averaging the peeling force and then dividing the force by the width of the sample. The adhesive force between a single microneedle and membrane was estimated by multiplying the adhesive force with the side length of the microneedle.

### Swelling test

The swelling pressure (*P*_swelling_) and the swelling ratio (*J*_gel_) of the hydrogel particles were measured by a customized pressure measuring device as shown in fig. S10. Briefly, 1 ml of dry hydrogel particles and a plate attached to the forcemeter (Mark-10, M4-5) were placed in a multihole tube glued to the bottom of the beaker. After adjusting the distance between the plate and the tube bottom, excess water was added to the beaker to initiate the swelling of the hydrogel. The force measured by the forcemeter then gradually increased and stabilized. The swelling pressure was calculated as the stabilized force divided by the area of the plate, and the swelling ratio (*J*_gel_) of the hydrogel was calculated as the product of the distance and the area of the plate divided by the initial hydrogel volume. By adjusting the distance between the plate and the tube bottom, the swelling pressure under different swelling ratios can be measured.

The swelling ratio of the microneedle robots (*J*_robot_) were tested using the drainage method. The microneedle robots under both dry and swollen state were put into a container of water, and their volume was quickly determined by measuring the increased volume of water. The *J*_robot_ was calculated as the fraction of the volume of the microneedle robot under swollen and dry state.

### Analysis of residual monomers and prepolymer

The residual percentage of monomer or prepolymer was analyzed using UPLC (Waters) using a C18 chromatographic column (1.7 μm, 2.1 mm by 50 mm). The obtained stretchable membranes and microneedle patches were firstly purified in 4 ml of phosphate-buffered saline (PBS) for 2 to 24 hours to determine the purification time. To quantify the residual monomers or prepolymers, the purified membrane or microneedle was incubated in 4 ml of renewed PBS for additional 24 hours. Gradient UPLC methods were developed (table S1). The concentration of the released monomer or prepolymer was determined at 205 nm on the basis of the calibration curve obtained from the standard solutions of each compound in varying concentrations. These measurements were performed in triplicate.

### Tissue penetration and retention test

The barbless microneedle patch and barbed microneedle patch with various *L*_barb_ and *n*_barb_ were used to characterize the penetration and retention effect. With a customized holder, the microneedle patch was linked to a 2-N forcemeter (Weidu Gage, SH-2) over the fresh intestinal tissue or 1% agar gel, which was fixed to a moving stage (Daheng Optics, GCD-301101 M) and moved upward at a rate of 0.1 mm s^−1^ until the substrate of the microneedle patch penetrated into the tissue or the gel. The penetration force against displacement was recorded, and the penetrated tissues were collected for histology observation to ensure the penetration depth. The penetration efficiency was calculated as the fraction of the microhole number and the microneedle number.

To measure the retraction force of the microneedle patches, they were pull out of the tissue or the gel and the retraction force against displacement was recorded. After 20 penetration-retraction cycles, the microneedles separated from the patch and retained in the tissues were numbered, and the retention efficiency was calculated as the fraction of the number of the retained microneedles and the total microneedle number.

The perforation force was tested using a single 32G stainless steel needle linked to a 2-N forcemeter (Weidu Gage, SH-2). The intestinal tissue was fixed to a moving stage (Daheng Optics, GCD-301101 M) and moved toward the needle at a rate of 0.1 mm s^−1^ until a force drop was observed. The maximum was denoted as the perforation force.

### In vitro drug release kinetics test

The microneedle patches loaded with RB-mPEG were put into a centrifugal tube and incubated in PBS at 37°C and shaken at 100 rpm. At specific time points, 200 μl of release samples was collected and replaced with same volume of fresh PBS. The concentration of released fluorescent dye and the release kinetics were determined and analyzed by an ultraviolet-visible spectrophotometer (Macylab instruments, UV-1800A).

To compare the drug delivery efficacy between different needles, barbless and barbed microneedle patches loaded with RB-mPEG were prepared, and the penetration-retraction cycles were repeated as described above. After 1-hour penetration-retraction cycles, the tissues with released RB-mPEG were ground in 1 ml of deionized water into a homogeneous mixture and the RB-mPEG was extracted with ethanol. After filtered, the concentration of released RB-mPEG in the tissues were analyzed by an ultraviolet-visible spectrophotometer (Macylab instruments, UV-1800A). The drug-delivery efficacy was calculated as the fraction of the mass of dye released in tissues and the initial loading.

### Degradation test

Materials that formed the robot including microneedles, membranes, and superabsorbent hydrogels were prepared as the same as above. To simulate the intestinal environment, materials were immersed in artificial intestinal fluids (pH = 6.8) and shaken in an incubator at 80 rpm and 37°C. At specific time points, material blocks were filtrated and weighed to measure their weight changes.

### In vitro biocompatibility test

CCK-8 assay was used to evaluate the biocompatibility of the materials used to fabricate the microneedle robot. The material extracts were obtained by incubating 0.1 g of the materials with 1 ml of DMEM containing 10% fetal bovine serum and 1% penicillin/streptomycin at 37°C for 24 hours. Mouse fibroblast cell line L929 were cultured in a 96-well plate for 12 hours to reach 80 to 90% confluency (*n* = 6). Cells were then treated with the extracts of various concentration and incubated at 37°C for 24 hours and assessed using CCK-8 kit. Absorbance at 450 nm was measured to determine cellular viability.

### Preparation of pressure sensor

To test the intestinal motility dynamics and the peristaltic contraction pressure, a series of capsule pressure sensor of different diameters were prepared as shown in fig. S14 (*27*). The capsule was prepared by 3D printing and installed with a built-in analog-to-digital converter to provide 24-bit pressure and temperature values. To differentiate ambient pressure and contraction pressure, each capsule-shaped pressure sensor was equipped with measurement units (sensor information) sealed with silicone at both the axial and radial ends, and the pressure difference was obtained as the intestinal peristaltic pressure (*P*_intestine_). Before in vivo test, the sensors were calibrated in depth-controlled water at body temperature (37° to 38°C).

### In vivo intestinal motility study

All animal tests were approved by and performed according to the animal care and ethics committee of Tsinghua University and Beijing Medical Service Biotechnology Co Ltd. Because of the anatomical and peristaltic similarities of the intestinal tract to humans, we chose Bama minipigs (three females; weight, ~30 to 50 kg) as the in vivo animal models. Before the studies, the pigs were placed on a liquid diet for 24 hours and fasted overnight to avoid the effects of food residues. With the assistance of an endoscopy system (GongJiang, Vet-100), the pressure sensor was directly delivered to the small intestine and the peristaltic pressure profiles was recorded. The frequency domain and the contraction pressure were analyzed as described (*27*).

### Micro–computed tomography imaging and x-ray fluoroscopy

X-ray contrast loaded microneedle robots were firstly prepared by loading 20–wt % barium sulfate in the stretchable membranes of the microneedle robots and confirmed by Micro–computed tomography (PerkinElmer, Quantum GX). Matrix size, 90 μm by 90 μm by 90 μm; scanning time, 4 min; current, 500 mA. To visualize the oral delivery and excretion routes, the contrast loaded microneedle robots were trans-orally delivered into the upper part of the minipigs’ esophagus (two females; weight, ~30 to 50 kg) and imaged every day using a clinical mobile C-arm imaging system (WDL, HHMC-100) until no microneedle robots were found in the digestive tracts of the minipigs.

### In vivo drug delivery efficacy evaluation

To evaluate the drug delivery efficacy, the insulin-loaded microneedle robots were delivered to the small intestine of Bama minipigs (10 females and 2 males; weight, ~30 to 50 kg) endoscopically and their interaction with dynamic intestine motility were recorded by an attached camera. After delivery, blood glucose levels were detected at the indicated time points using a OneTouch glucose monitor. In the meanwhile, blood was collected from the jugular vein and the serum was separated to determine the insulin levels using a Human insulin ELISA kit (i-presci Scientific).

### Histological analysis

Intestinal tissues were harvested at the site of penetration from minipigs (two females; weight, ~30 to 50 kg) after microneedle robot deployment. Tissues were then fixed in 10% buffered formalin for 48 hours, embedded in paraffin, and processed using a microtome (Leica). Hematoxylin and eosin staining method was performed to evaluate tissue penetration, inflammation, and recovery.

### Bioavailability calculations

The relative bioavailability was calculated as the fraction of the area under the plasma concentration-time curve (AUC) of test dosage and subcutaneous administration

$$\text{Relative bioavailability}=\frac{{\left({\mathrm{AUC}}_{T}\right)}_{\text{test}}∕D_{\text{test}}}{{\left({\mathrm{AUC}}_{T}\right)}_{S.C.}∕D_{S.C.}}\times 100\%$$

where (*AUC_T_*)_test_ and (*AUC_T_*)_S.C._ are the total areas under the plasma concentration-time curves following the administration of the test dosage form and subcutaneous injection, and *D*_test_ and *D*_S.C._ are the size of the dose of the drug administered by the test dosage form and subcutaneous injection, respectively.

### Statistical analysis

All of the results presented are means ± SD. Statistical analysis was performed using two-sided Student’s *t* test and two-way ANOVA test with the software Graphpad Prism. The differences between experimental groups and control groups were considered significant at *P* < 0.05.

## Supplementary Materials

### This PDF file includes:

Notes S1 and S2 Figs. S1 to S23 Tables S1 to S4 Legends for movies S1 to S3 References
