# Supplementary material for: Pain-free oral delivery of biologic drugs using intestinal peristalsis–actuated microneedle robots
Source: Sci Adv. 2024 Jan 5;10(1):eadj7067. doi: 10.1126/sciadv.adj7067 (PMC10776013; doi:10.1126/sciadv.adj7067)
Supplement: Supplementary file 1 — Notes S1 and S2 Figs. S1 to S23 Tables S1 to S4 Legends for movies S1 to S3 References [file sciadv.adj7067_sm.pdf]

Science Advances
AAAS

Supplementary Materials for

Pain-free oral delivery of biologic drugs using intestinal peristalsis–actuated microneedle robots

Xize Gao *et al.*

Corresponding author: Mingjun Zhang, mjzhang@tsinghua.edu.cn; Jing Xu, jingxu@tsinghua.edu.cn

Sci. Adv. **10**, eadj7067 (2024)
DOI: 10.1126/sciadv.adj7067

The PDF file includes:

Notes S1 and S2
Figs. S1 to S23
Tables S1 to S4
Legends for movies S1 to S3
References

Other Supplementary Material for this manuscript includes the following:

Movies S1 to S3

SUPPLEMENTARY NOTE 1

Analysis of the peristaltic force acting on microneedles

As illustrated in **Fig. S1**, peristaltic contractions acting on the pressure sensor cause deformations of the intestinal wall. When the capsule pressure sensor is positioned inside the intestinal lumen, it experiences the contractions directly. As the intestinal wall contracts, the intestine generates pressure on the sensor, thereby creating a flattened contact surface (**Fig. S1A**). Once the sensor is substituted with the microneedle robot, the intestinal wall will act on the defined shape. In case of sparse microneedles, the intestinal wall depresses between two adjacent microneedles during contractions (**Fig. S1B**).

The proposed microneedle robot utilizes densely packed arrays with a needle-to-needle distance of approximately 1.2 mm, much smaller than the length of the contractive intestine ranging between 70-100 mm (*58*). As a result, the intestinal wall conforms around the dense array of microneedles, generating a flat contact surface (**Fig. S1C**).

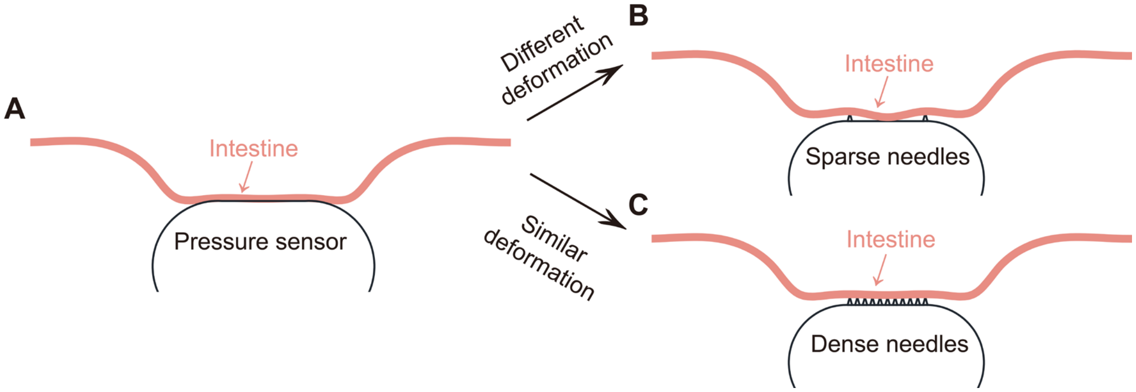

Fig. S1. Deformation of the intestinal wall when contacting with the pressure sensor and the microneedle robot. (A) The pressure sensor. (B) The microneedle robot with sparse needles. (C) The microneedle robot with dense needles.

If the intestinal peristaltic strength is uniform, the peristaltic contraction force acting on the microneedle robot (F_{robot}) and on the sensor (F_{sensor}) is similar, because the deformations of the intestinal wall are similar.

$$F_{robot} \approx F_{sensor} \tag{S1}$$

The contraction force acting on the sensor can be then expressed as

$$F_{sensor} = P_{sensor} \cdot S_{sensor} \tag{S2}$$

where P_{sensor} is the pressure measured by the sensor with the same diameter as the microneedle robot and S_{sensor} is the area of the pressure sensor. Assume the number of microneedles on S_{sensor} is N, the contraction force applied on each microneedle can be calculated as

$$F_{needle} = \frac{F_{robot}}{N} \approx \frac{P_{sensor} \cdot S_{sensor}}{N} \tag{S3}$$

SUPPLEMENTARY NOTE 2

Analysis of the force balance between the hydrogel swelling pressure and the membrane tension

The cross section of the microneedle robot is close to circular at the swelling equilibrium state, so it can be simplified as a cylindrical barrel expandable as shown in **Fig. S2**. Generally, the robot's length along the Z-axis is relatively constant due to the presence of the lip, hence we can assume that the deformation of the microneedle robot is within the XY-plane.

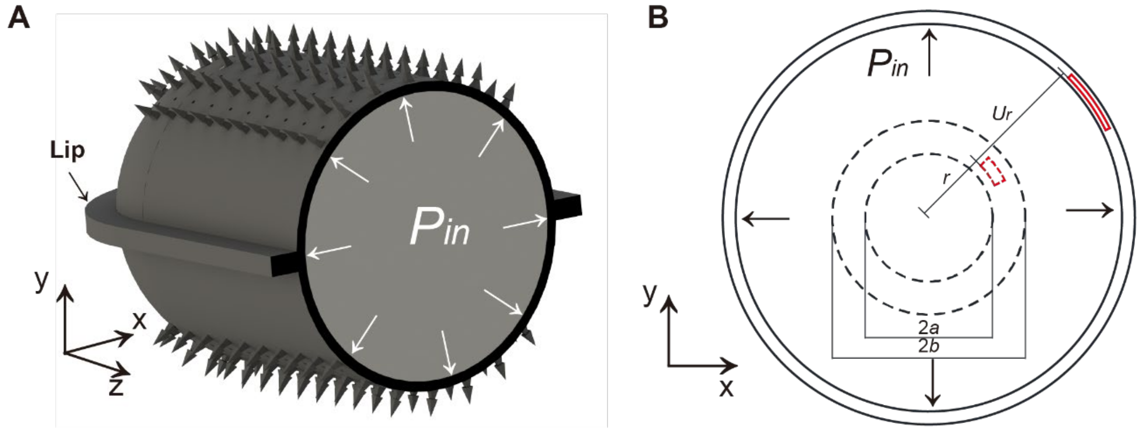

Fig. S2. Schematic of the microneedle robot (A) and the deformation in the cross-section XY-plane during swelling (B). The black dashed annulus is the wall of the microneedle robot in the dry state and the red dashed area is a micro unit chosen on the wall. At the swelling equilibrium state, the wall of the microneedle robot will be stretched into the black continuous annulus and the micro unit will be stretched into the red continuous area.

Assume the inner radius of the microneedle robot is a and the outer radius is b in the dry state. The radial displacement U_r of a micro unit chosen on the wall of the microneedle robot can be calculated by the equations of Lamé (*59*).

$$U_r = Ar + \frac{B}{r} \tag{S4}$$

and the constants A and B are

$$\begin{cases} A = \dfrac{(1+\nu)(1-2\nu)}{E}\dfrac{a^2}{b^2-a^2}P_{in} \\ B = \dfrac{1+\nu}{E}\dfrac{a^2b^2}{b^2-a^2}P_{in} \end{cases} \tag{S5}$$

where P_{in} is the internal pressure, E is the elastic modulus and ν is the Poisson's ratio of the membrane. Since the microneedle robot has only two dimensions to swell, the swelling ratio of the microneedle robot (J_{robot}) can be expressed as

$$J_{robot} = \lambda^2 = (\frac{U_b}{b} + 1)^2 = (A + 1 + \frac{B}{b^2})^2 \tag{S6}$$

where λ is the radial stretch ratio, and U_b is the radial displacement of the outer surface. From **Eqns. S5** and **S6**, we have

$$P_{in} = \frac{2E}{1-\nu^2}\frac{b^2-a^2}{a^2}\left(J_{robot}{}^{\frac{1}{2}}-1\right) \quad (S7)$$

For the microneedle robot, the internal pressure is the swelling pressure ($P_{swelling}$) of the hydrogel particles. Assume an ideal elastomeric gel model, the swelling capability of hydrogel depends on the balance between the osmotic stress ($\pi_{osmotic}$) and the elastic restoring stress ($\sigma_{elastic}$) (*39*).

Due to the wrapping of the membrane, the swelling ratio of the hydrogel at the swelling equilibrium state is much smaller than that in the bare case. As a result, the osmotic stress is much greater than the elastic restoring stress.

$$P_{swelling} = \pi_{osmotic} - \sigma_{elastic} \approx \pi_{osmotic} \quad (S8)$$

After introducing the Flory-Huggins model into the model, osmotic stress can be expressed as

$$\pi_{osmotic} = -\frac{kT}{\Omega}\left[\frac{1}{J_{gel}} + \log\left(1-\frac{1}{J_{gel}}\right) - \frac{1}{\alpha J_{gel}} + \frac{\chi}{J_{gel}{}^2}\right] \quad (S9)$$

where J_{gel} is the swelling ratio of the hydrogel particles, kT, Ω, α and χ are respectively the temperature in the unit of energy, the volume per solvent molecule, the volume per polymer chain divided by the volume per solvent molecule, a dimensionless measure of the enthalpy of mixing. So, the swelling pressure can be obtained from **Eqns. S8** and **S9**.

$$P_{swelling} = -\frac{kT}{\Omega}\left[\frac{1}{J_{gel}} + \log\left(1-\frac{1}{J_{gel}}\right) - \frac{1}{\alpha J_{gel}} + \frac{\chi}{J_{gel}{}^2}\right] \quad (S10)$$

Assume the volume of the microneedle robot in dry state is $V_{0,robot}$, the volume of the hydrogel particles in dry state is $V_{0,gel}$, the ratio of $V_{0,gel}$ and $V_{0,robot}$ is the filling ratio (η). The volumes of the microneedle robot and the hydrogel particles are the same (V_1) at the swelling equilibrium state. The swelling ratio of the hydrogel particles can be expressed as

$$J_{gel} = \frac{V_1}{V_{0,gel}} = \frac{V_1}{\eta V_{0,robot}} = \frac{J_{robot}}{\eta} \quad (S11)$$

Inserting **Eqn. S11** into **S10**, we have

$$P_{swelling} = -\frac{kT}{\Omega}\left[\frac{\eta}{J_{robot}} + \log\left(1-\frac{\eta}{J_{robot}}\right) - \frac{\eta}{\alpha J_{robot}} + \frac{\eta\chi}{J_{robot}{}^2}\right] \quad (S12)$$

Combining **Eqns. S7** and **S12**, we have

$$\frac{2E}{1-\nu^2}\frac{b^2-a^2}{a^2}\left(J_{robot}{}^{\frac{1}{2}}-1\right) = -\frac{kT}{\Omega}\left[\frac{\eta}{J_{robot}} + \log\left(1-\frac{\eta}{J_{robot}}\right) - \frac{\eta}{\alpha J_{robot}} + \frac{\eta\chi}{J_{robot}{}^2}\right] \quad (S13)$$

Eqn. S13 indicates that when the initial size of the microneedle robot and hydrogel particles are assigned, the values for parameters a, b, kT, Ω, α and χ remain constant. Consequently, the swelling ratio of the microneedle robot is predominantly influenced by the elastic modulus of the membrane materials and the filling ratio. As shown in **Fig. S3**, assuming the elastic modulus of the membrane to be 64 kPa, the intersections can be used to determine the theoretical swelling ratio for a set of filling ratios. Similarly, by setting the filling ratio to 25.0%, the intersections will determine the theoretical swelling ratio for a set of membrane elastic moduli.

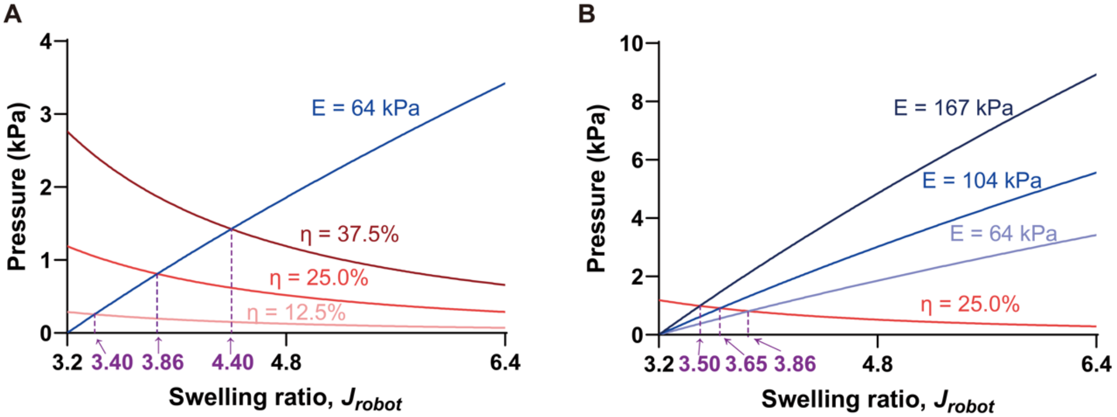

Fig. S3. Calculated swelling ratio of the microneedle robot with various filling ratios (A) and elastic moduli (B) at the swelling equilibrium state. If we place a dry microneedle robot without hydrogel particles in fluids, the microneedle robot will swell to 3.2 times to its initial dry size due to the swelling of the membrane. Throughout this process, the wall of the microneedle robot remains tension-free. Only when the swelling ratio is greater than 3.2, the membrane of the microneedle robot starts to stretch and the membrane tension starts to increase from a baseline of zero.

SUPPLEMENTARY FIGURES

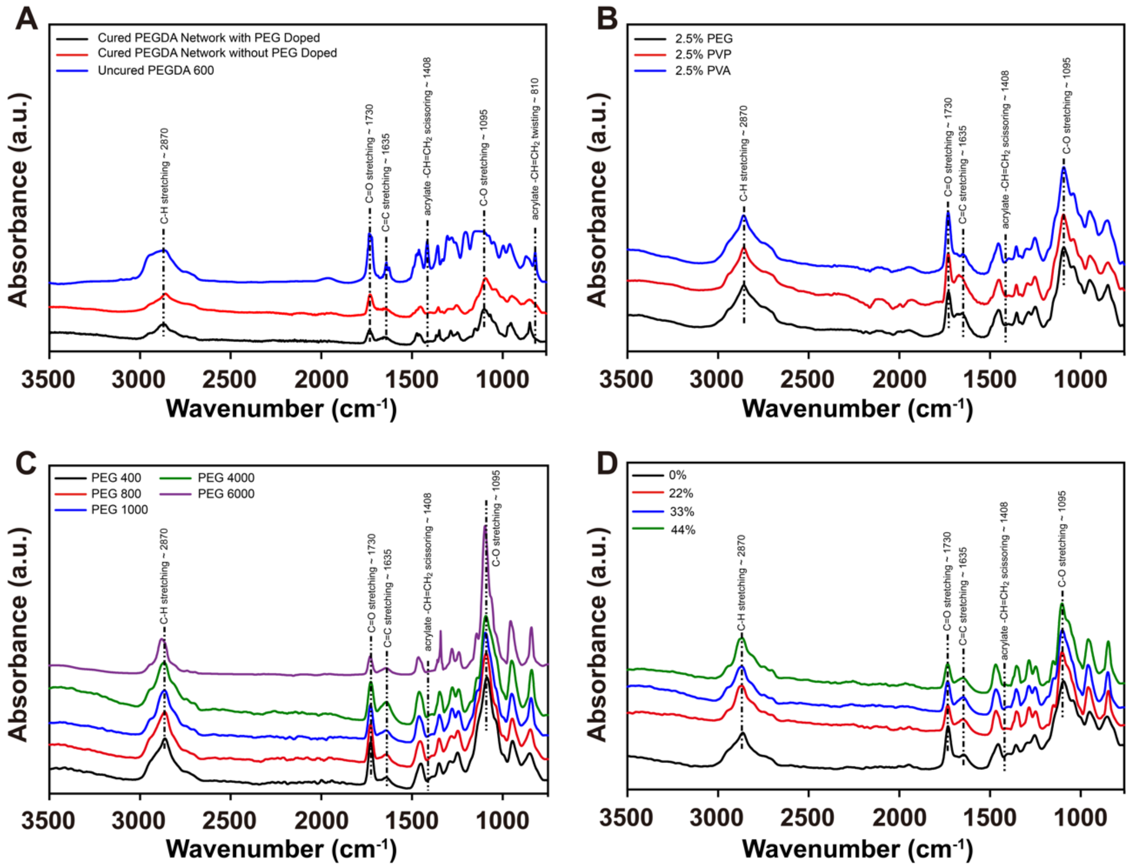

Fig. S4. ATR-FTIR spectra of the PEGDA-dopant hydrogels. (A) Representative ATR-FITR spectra of uncured PEGDA, cured PEGDA with and without PEG. The bands identified at 1635, 1408, and 810 cm^{-1} are related to C=C stretching vibration, in-plane scissoring vibration of -CH acrylate end groups, and out-of-plane twisting vibration of acrylate $-CH=CH_2$, respectively. The peak intensity at 1635 cm^{-1} in the spectra corresponds to the number of residual carbon-carbon double bonds which allow for further photopolymerization. After curing, the peaks at 1408 cm^{-1} and 810 cm^{-1} vanished, while the peak at 1635 cm^{-1} diminished, indicating the partial conversion of C=C bonds into C–C bonds. It was observed that the cured PEGDA network doped with PEG exhibited a higher residual percentage of double bonds compared to the PEG-free group. (B) Representative ATR-FITR spectra of PEGDA hydrogels diluted with different polymers. (C-D) As the molecular weight or the percentage of PEG increased, an increase of the signals of C=C in the networks was observed. However, with further increases in the molecular weight or the percentage of PEG, PEG reached the overlap concentration and formed dense brushes on the surface of PEGDA-rich domains. This phenomenon affected the detection of PEGDA and resulted in a decreased signal for double bonds (60). These data supported the "inverted U" pattern of the connection force between microneedles and membranes in Figure 2H from a component perspective. All results were replicated at least three times.

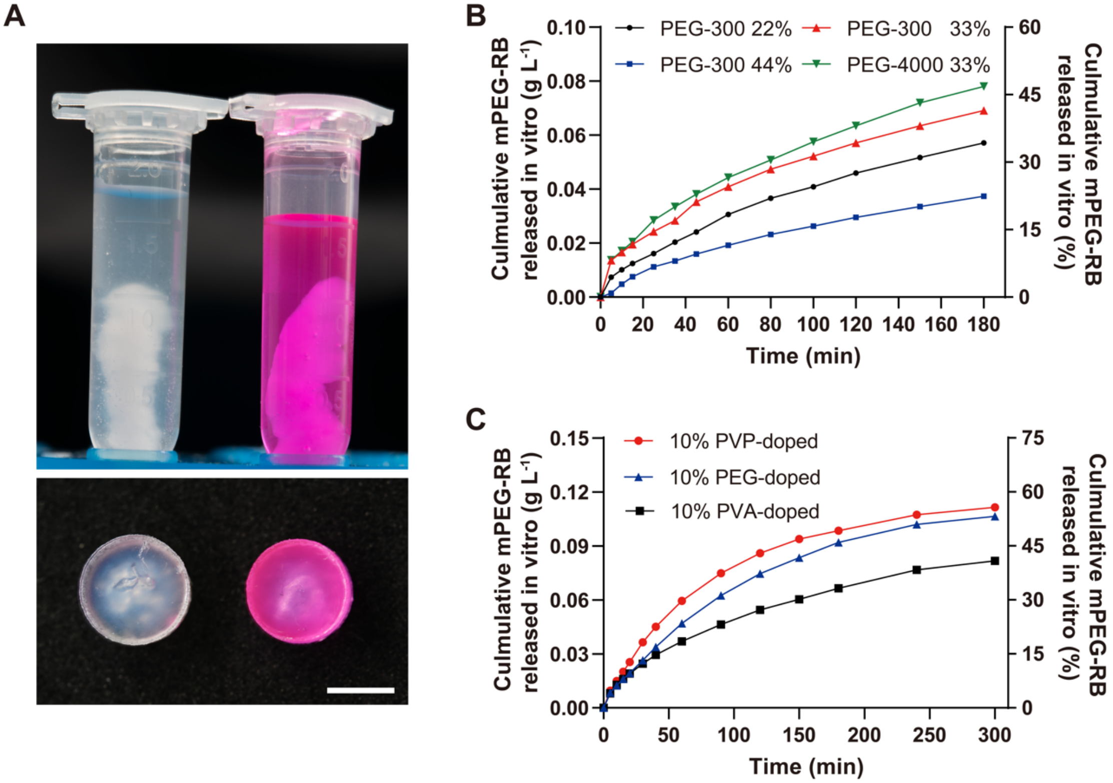

Fig. S5. Drug release of the PEGDA-polymer mixtures. (A) Phase separation phenomenon observed in the mixture of PEGDA and PVA aqueous solutions (top) and PEGDA-PVA hydrogel (bottom) without (left) and with (right) the presence of rhodamine-labelled mPEG-5000. Scale bar, 5 mm. (B) *In vitro* accumulated release of rhodamine labelled with mPEG-5000 of the PEGDA-PEG hydrogel under variable PEG contents. (C) *In vitro* accumulated release of the rhodamine labelled mPEG-5000 of PEGDA-PEG, PEGDA-PVA and PEGDA-PVP hydrogel subjected to 10% doping.

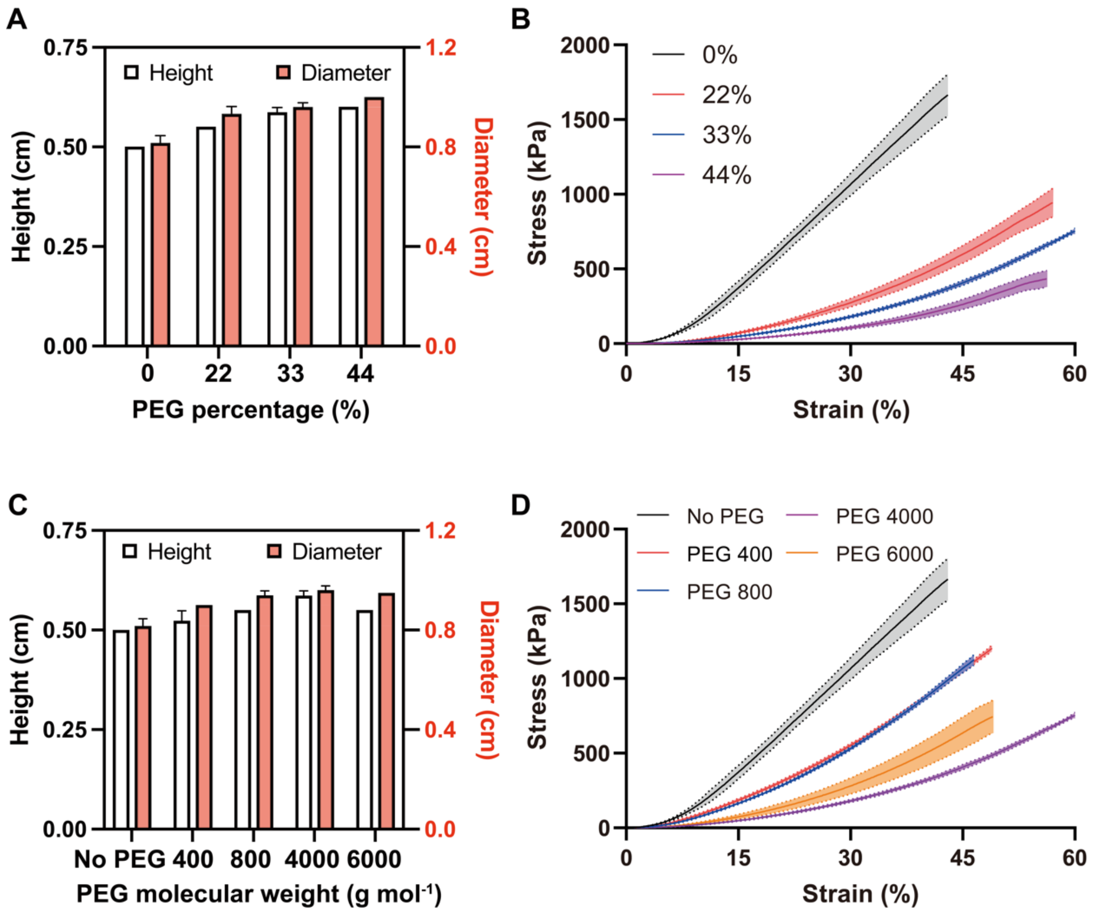

Fig. S6. Influences of the introduced PEG on the swelling properties and mechanical strength of the PEGDA/PEG hydrogels. (A and B) Swelling behavior and mechanical strength of PEGDA/PEG hydrogels with various PEG mass percentages. The volume ratio of PEGDA was maintained as 33% and the PEG molecular weight was maintained as 4000 g mol^{-1}. N = 3 technical replicates. (C and D) Swelling behavior and mechanical strength of PEGDA/PEG hydrogels with various PEG molecular weights. The volume ratio of PEGDA and the mass percentage of PEG were maintained as 33%. The original height and diameter of hydrogel column is 0.5 cm and 0.8 cm, respectively. N = 3 technical replicates.

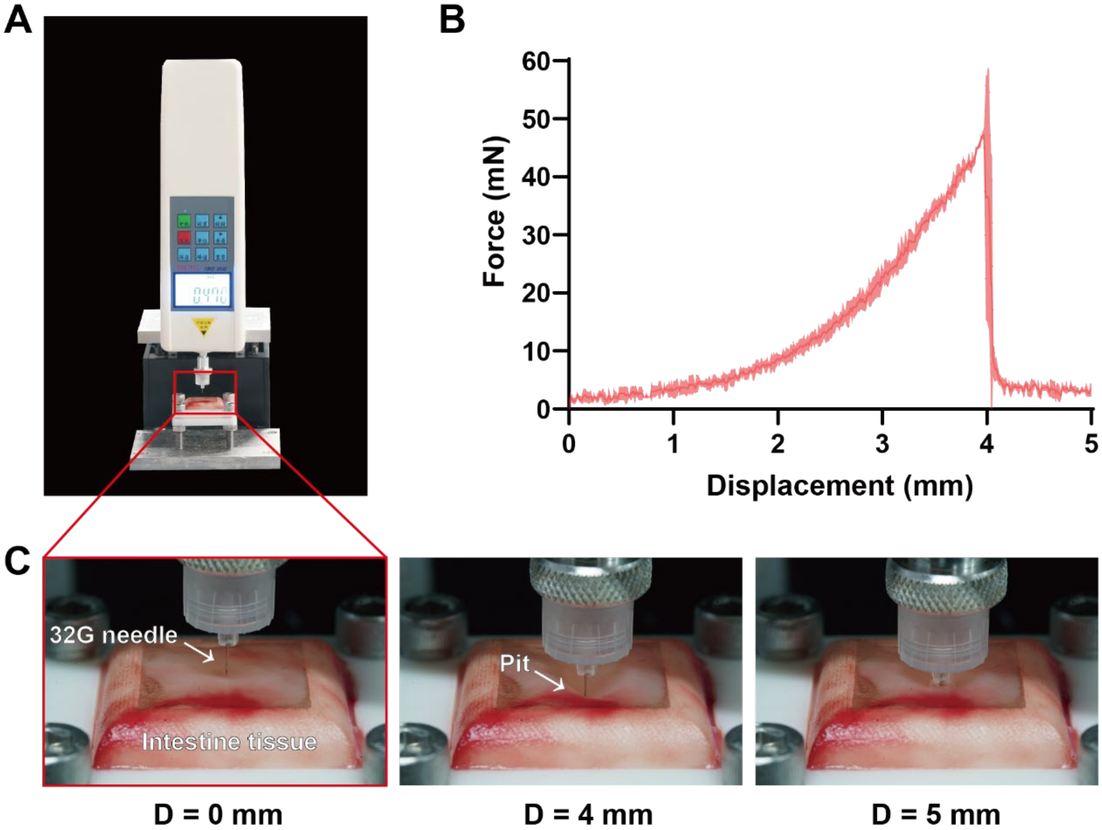

Fig. S7. Perforation test on ex vivo porcine intestinal tissue and the test setup. (A) Perforation test setup. (B) Force-displacement curves of perforation test. N = 3 technical replicates. The force drop denotes the perforation. (C) Photographs of the *ex vivo* minipig intestine before, during and after the perforation.

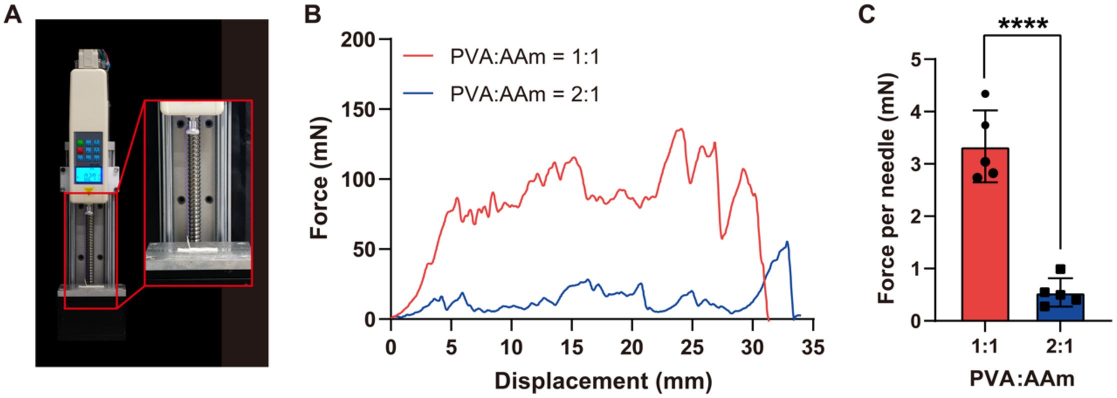

Fig. S8. Influences of the AAm contents on the adhesive force of the microneedle-membrane interface and the test setup. (A) Photograph of the 90º peeling test. (B) Representative adhesive force between the microneedles and membrane with various AAm contents tested by the 90º peeling test. N = 5 technical replicates. (C) Calculated adhesive force per needle at the microneedle-membrane interface, two-tailed student's t-test, ****P<0.0001.

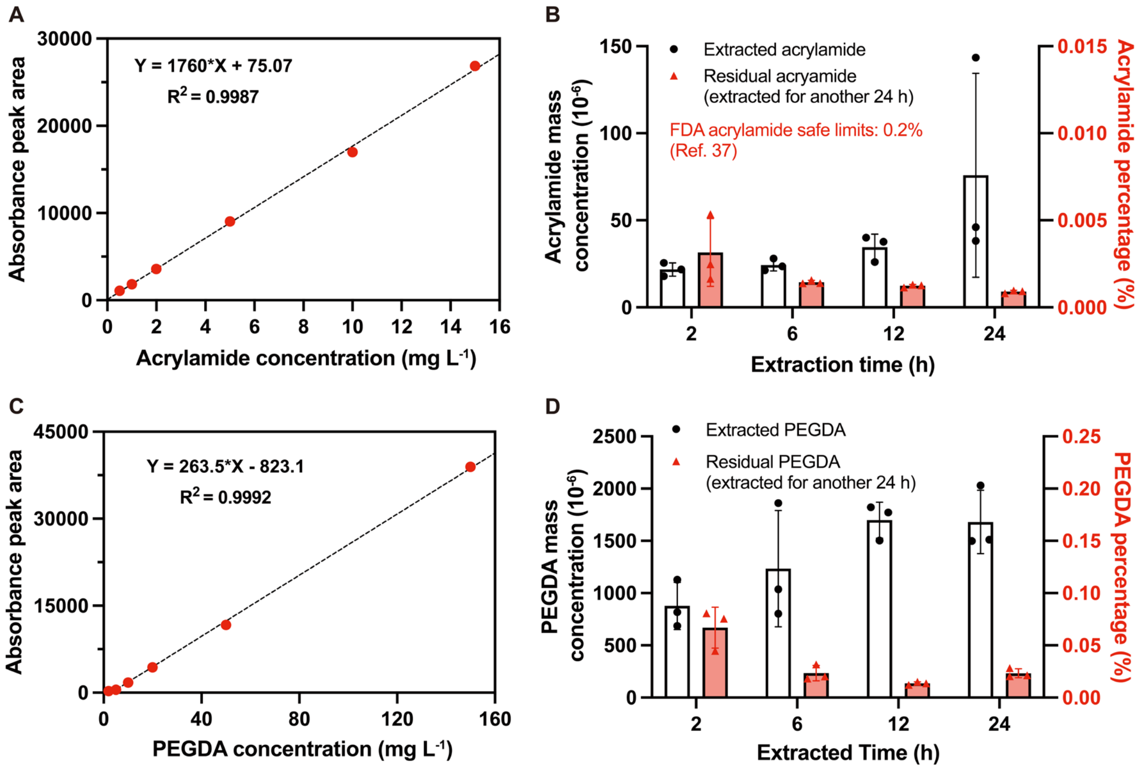

Fig. S9. Quantification of residual monomer in the microneedle robot by UPLC. (A) Standard calibration curve of acrylamide for UPLC. (B) UPLC characterization of the extraction solution from PVA/PAAm membranes extracted for different durations (black dots). Red triangles represented the residual acrylamide extracted for additional 24 hours. N = 3 technical replicates. The percentage of residual acrylamide was far below the FDA safe limits in all the test results, which indicated that the PVA/PAAm membrane did not require purification before use. (C) Standard calibration curve of PEGDA for UPLC. (D) UPLC characterization of the extraction solution from PEGDA/PEG microneedles extracted for different durations (black dots). Red triangles represented the residual PEGDA extracted for additional 24 hours. N = 3 technical replicates.

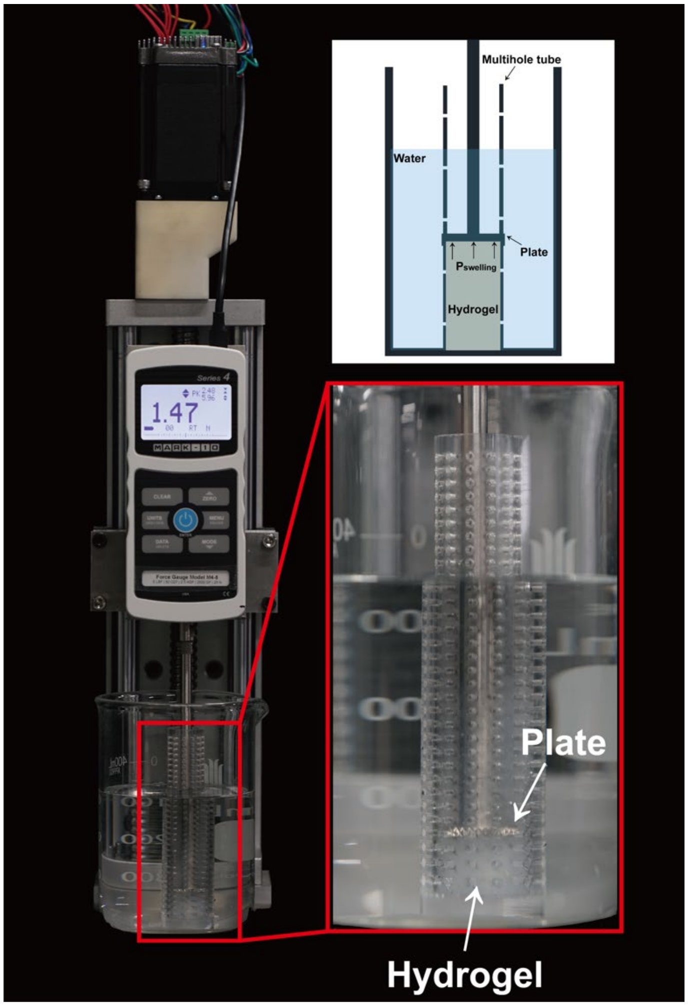

Fig. S10. Setup for the hydrogel swelling pressure test.

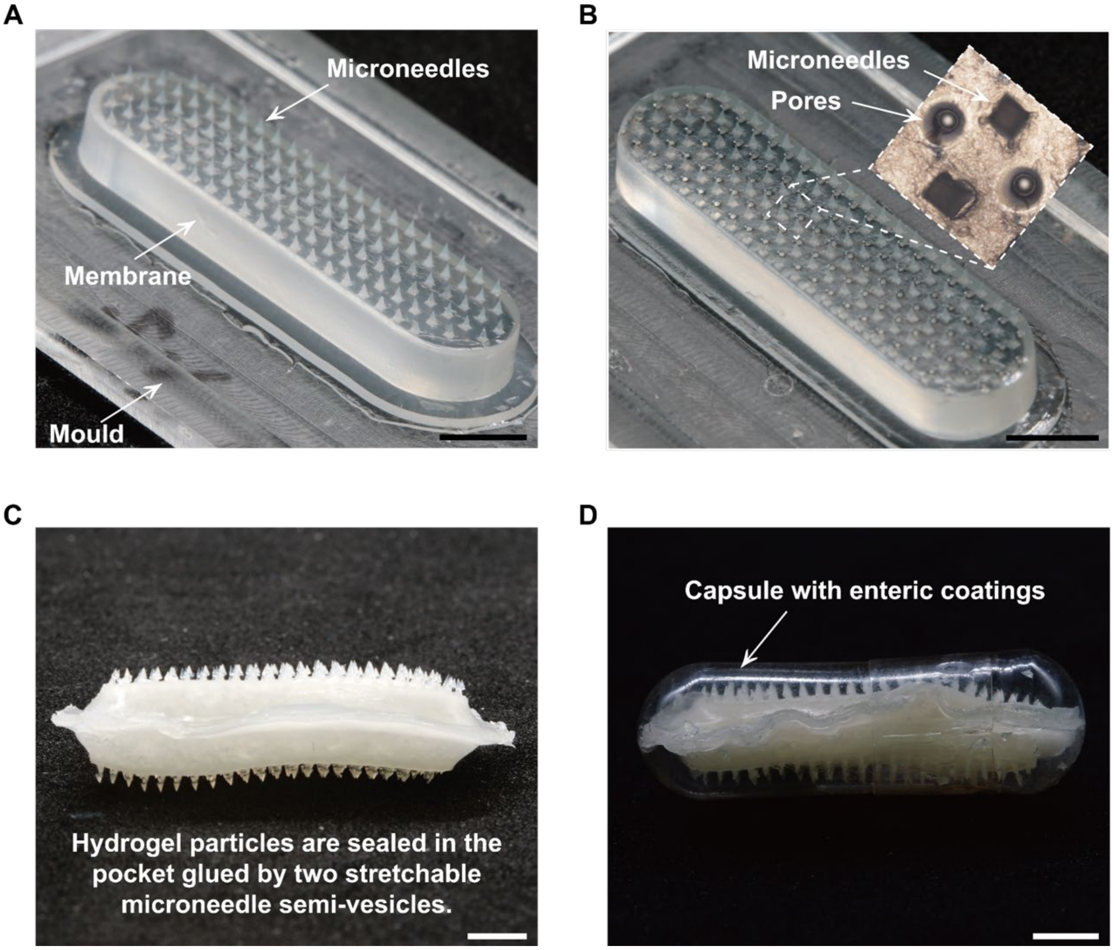

Fig. S11. Assembling of the microneedle robots. (A) Preparation of the stretchable microneedle semi-vesicle. (B) Laser boring of the stretchable microneedle semi-vesicle. (C) Hydrogel particles loading and sealing. (D) A microneedle robot encapsulated by a capsule with enteric coatings. Scale bars, 5 mm.

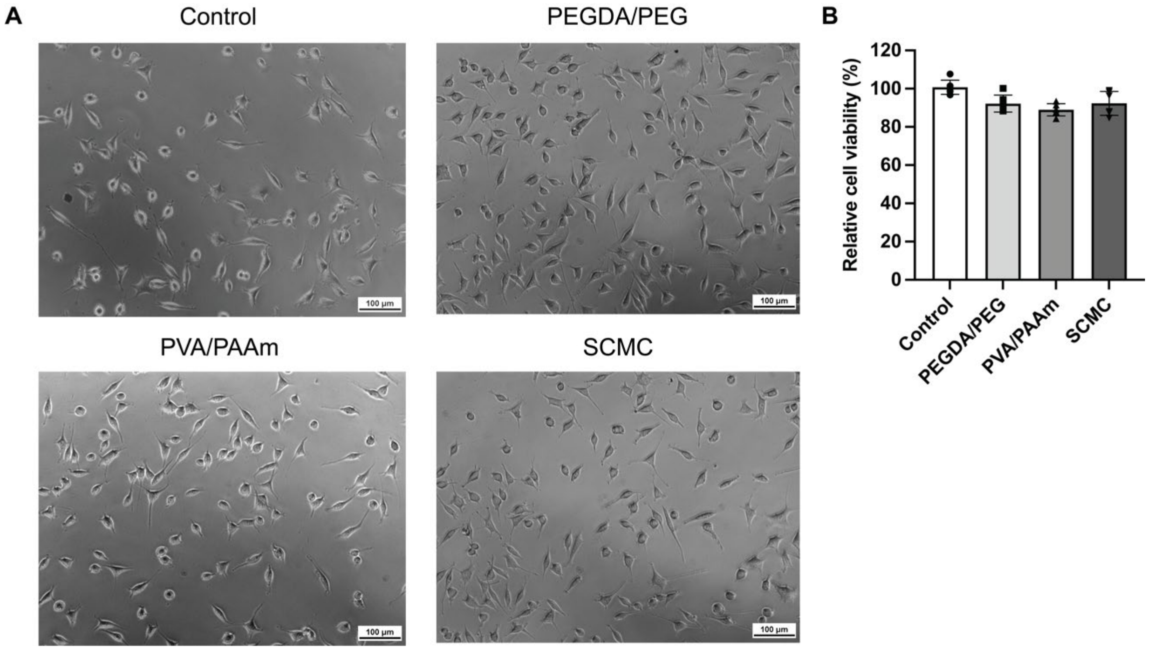

Fig. S12. *In vitro* biocompatibility assay of the materials used to fabricate the microneedle robot. (A) Representative microscopic photographs and (B) relative cell viability of mouse fibroblast cells after 24 hours of co-culture with the material extracts characterized using the CCK-8 method. N = 6 technical replicates.

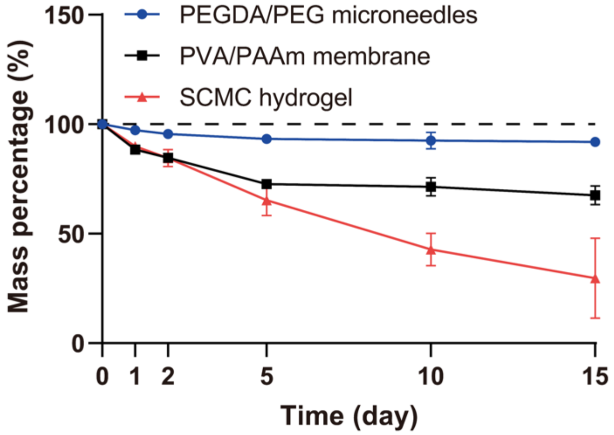

Fig. S13. Degradation curves of the microneedles, membrane and hydrogel in simulated intestinal fluids. The curves indicate that the materials used to construct the microneedle robot are biodegradable. N = 3 technical replicates.

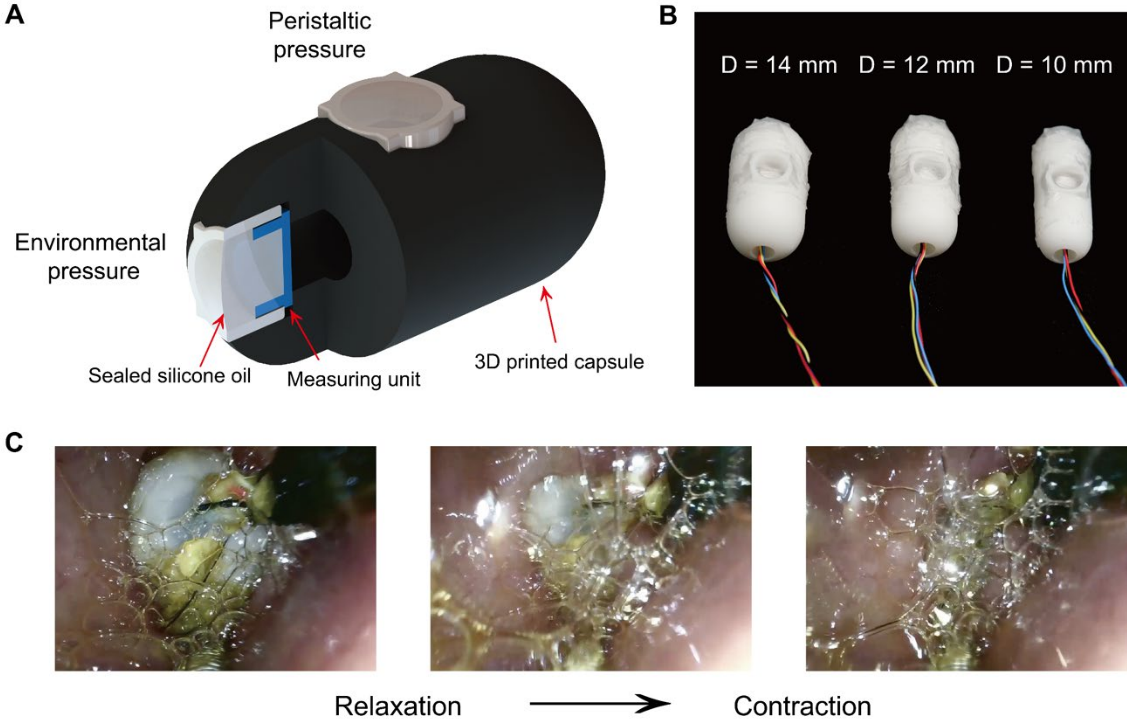

Fig. S14. Intestine manometry setup. (A) Schematic diagram of the pressure sensor. (B) Photograph of the pressure sensors with diameters of 10 mm, 12 mm and 14 mm. (C) Gastroscopic images of the pressure sensor in the intestine. As the intestine contracts, the pressure sensor is squeezed and the measured pressure gradually increases.

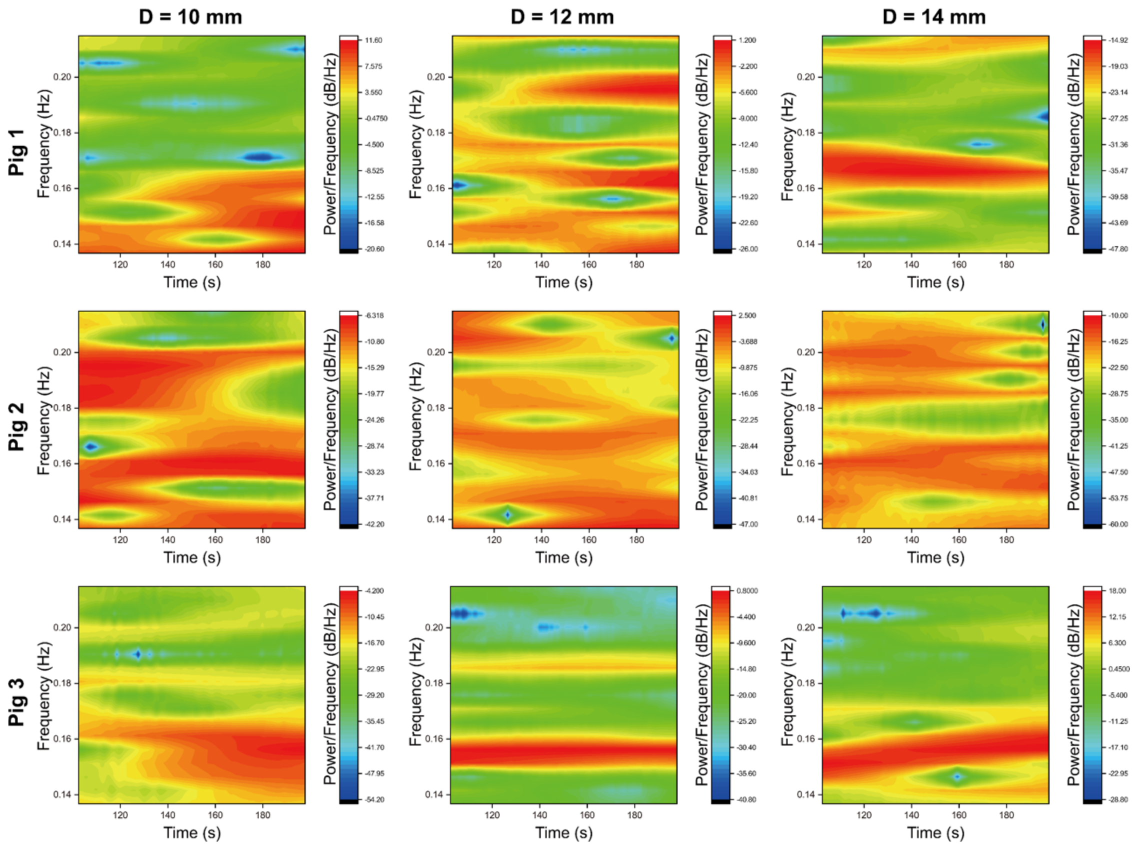

Fig. S15. Time-frequency graphs of the intestinal peristalsis measured by pressure sensors with diameters of 10 mm, 12 mm and 14 mm in three minipigs *in vivo*. The red regions around 0.167 Hz can be observed in all graphs, indicating that the pressure signals measured in three minipigs using pressure sensors with three diameters all have the primary frequency at about 0.167 Hz, which is corresponds to 10 cpm.

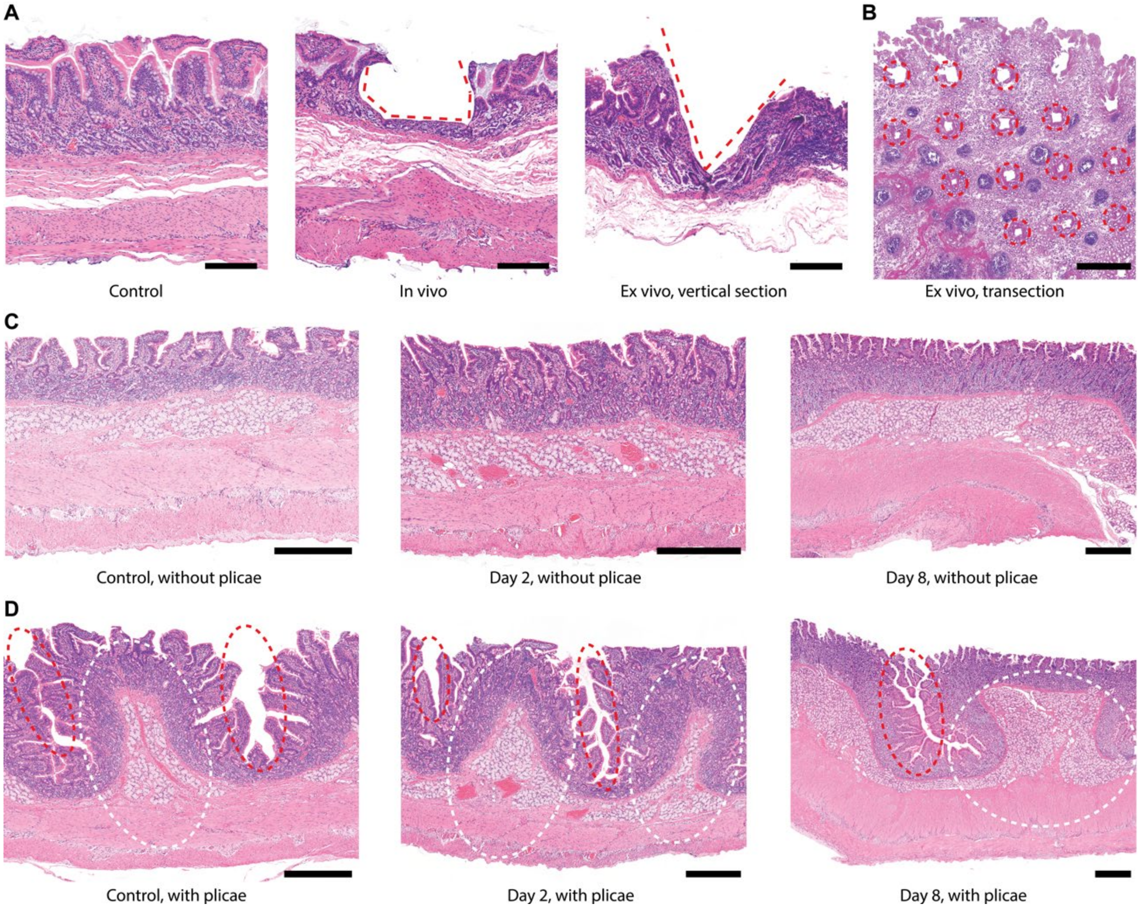

Fig. S16. Histological analysis of tissue penetration, inflammation and recovery on minipig intestinal tissues. (A) Representative hematoxylin-eosin staining images showing the tissue penetration after microneedle robot delivery *in vivo* and *ex vivo* microneedle patch pressing. The images indicate that the microneedle robot achieved effective tissue penetration *in vivo* using intestinal peristalsis comparable to *ex vivo* pressing. No intestinal perforation or bleeding was caused by the microneedle penetration. Red dashed lines indicated the penetration. Scale bar, 0.2 mm. (B) Representative transections of *ex vivo* intestinal tissues penetrated by the microneedle array. Red dashed circles indicated the pinholes. Scale bar, 1.0 mm. (C-D) Representative histology samples harvested from the injection sites with (D) or without (C) plicae on Day 2 and Day 8. White dashed circles indicated the intestinal plicae. Red dashed circles indicated the intervals between plicae rather than the injection holes. No difference was observed among the tissue sections from the control group and those taken one day or one week after the injection, which indicated limited inflammation and fast recovery of the intestinal tissues following *in vivo* microneedle penetration. Scale bar, 0.5 mm.

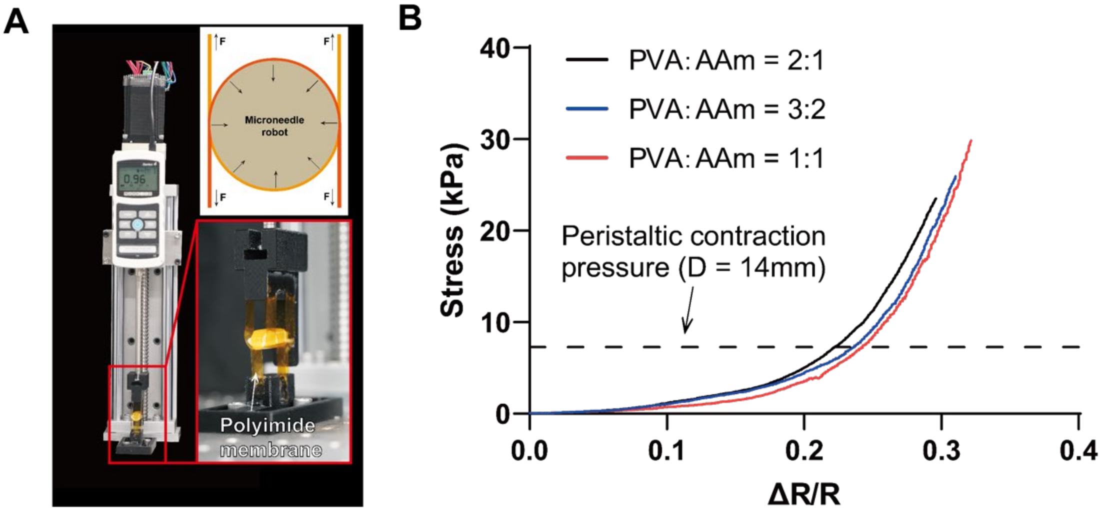

Fig. S17. Influences of membrane tension on the compression resistance of the microneedle robot and the circular compressive test setup. (A) Photograph of the circular compression test setup. (B) Representative stress-strain (ΔR/R) curves of the microneedle robots with various membrane moduli characterized by circular compression test. The curves indicate that all the microneedle robots could endure up to 25 kPa of circular compressional pressure before rupture, which is much larger than the peristaltic pressure of the small intestine which confirms that the microneedle robot is robust enough to keep intact under peristaltic contraction and support the needles to penetrate into the intestine. With the increase of the ratio of PVA to AAm, the strength of the microneedle robot increases slightly.

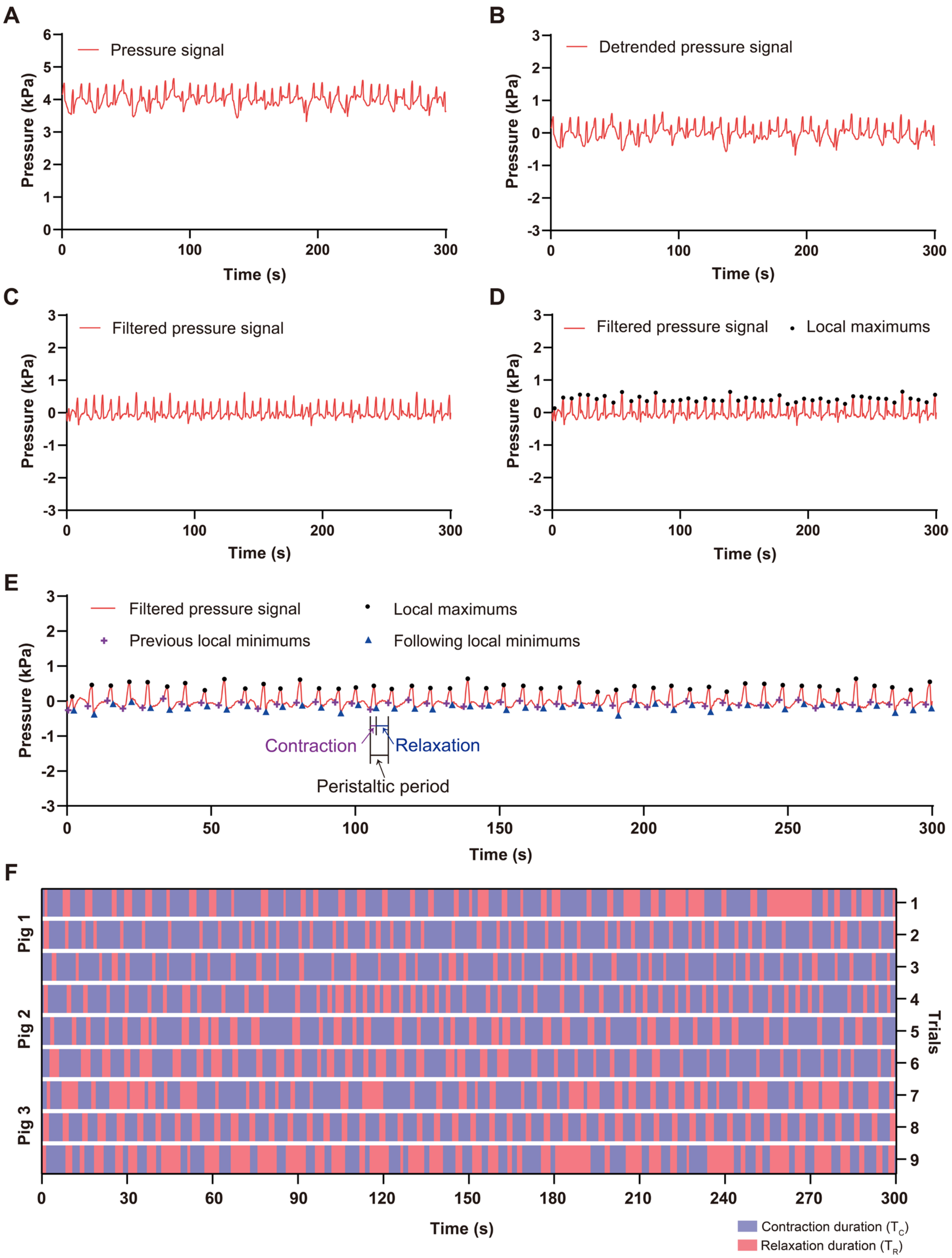

Fig. S18. Analysis of the contraction and relaxation durations of peristalsis. (A-E) Analysis procedure taking the pressure signals obtained from minipig 3 with a pressure sensor of 12 mm in diameter as an example. To calculate the contraction and relaxation time in a whole peristaltic period, we used MATLAB to process the peristaltic pressure signals (A). The MATLAB's detrend function was used to subtract offset contained in the pressure signal first (B). And then the

bandpass function filtered the pressure signals with a passband frequency range from 0.04 Hz to 0.5 Hz (C), which can filter out the signals induced by respiration and heart rate of the pig. After filtration, the islocalmax function was used to find local maximums of the filtered signal (D). Having local maximums, the previous and following local minimums were found (E). We defined the duration between two adjacent preceding local minima as a complete peristaltic cycle. Generally, a peristaltic cycle encompasses two local minima and one local maximum. We assigned the intervals between two local minima as the contraction duration, while the remaining time defined as the relaxation duration. (F) Results of 300 seconds of intestinal peristaltic contraction and relaxation alternations on three minipigs. The contraction duration usually accounts for about 32.3% of a peristaltic period while the relaxation duration making up the remaining 67.7%, which is consistent with the previous study (*22*).

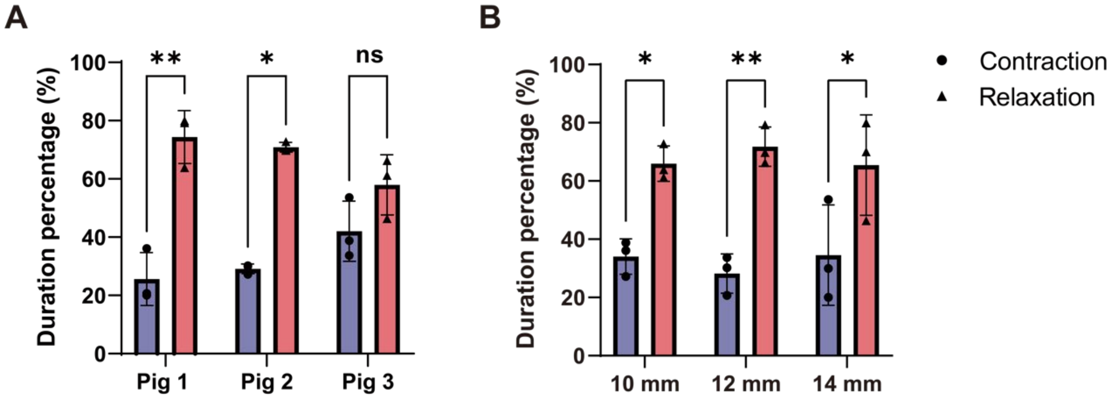

Fig. S19. Statistical analysis of the duration differences between the contraction and relaxation on different minipigs (A) and tested by pressure sensor of different diameters (B). N = 3 technical replicates for each minipig. Two-way ANOVA test, *P < 0.05, **P < 0.01, ns, nonsignificant. The data confirms that the relaxation duration is much longer than the contraction duration.

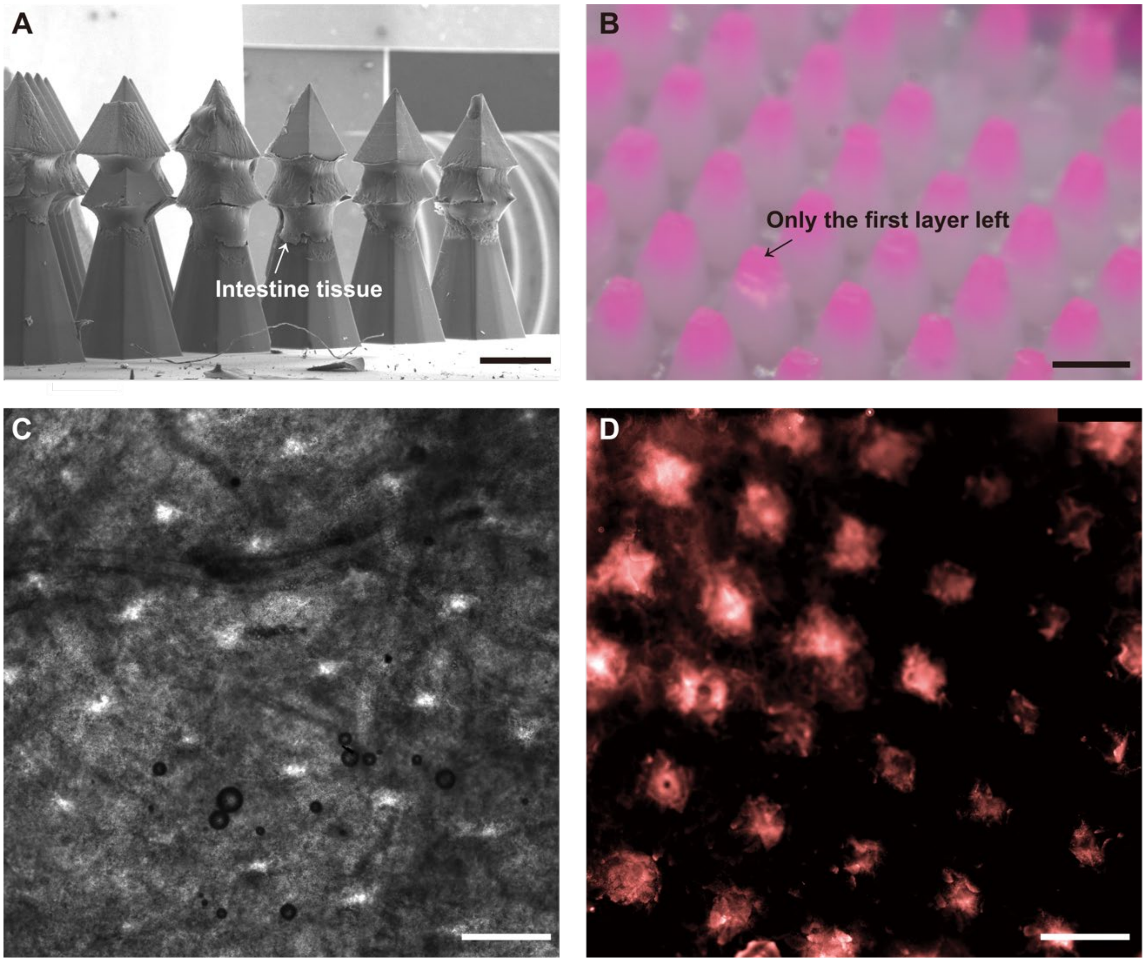

Fig. S20. Barbed microneedles improved drug delivery efficacy. (A) SEM image of the microneedle patches (n_{barb} = 2) after retraction from the *ex vivo* minipig intestine tissue. Scale bar, 500 μm. The image indicates an interlocking effect between the barbed structure and the fibrous tissue as the microneedles retract from the intestine. (B) Photograph of the barbed microneedle patch after retracted from a minipig intestine tissue *ex vivo*, showing the separation of barbs from the first layer of the microneedle. Scale bar, 1 mm. (C-D) Bright-field (C) and fluorescent microscopic images (D) showing the delivery of fluorescent dye into intestine tissues of a minipig *ex vivo*. Scale bar, 1 mm.

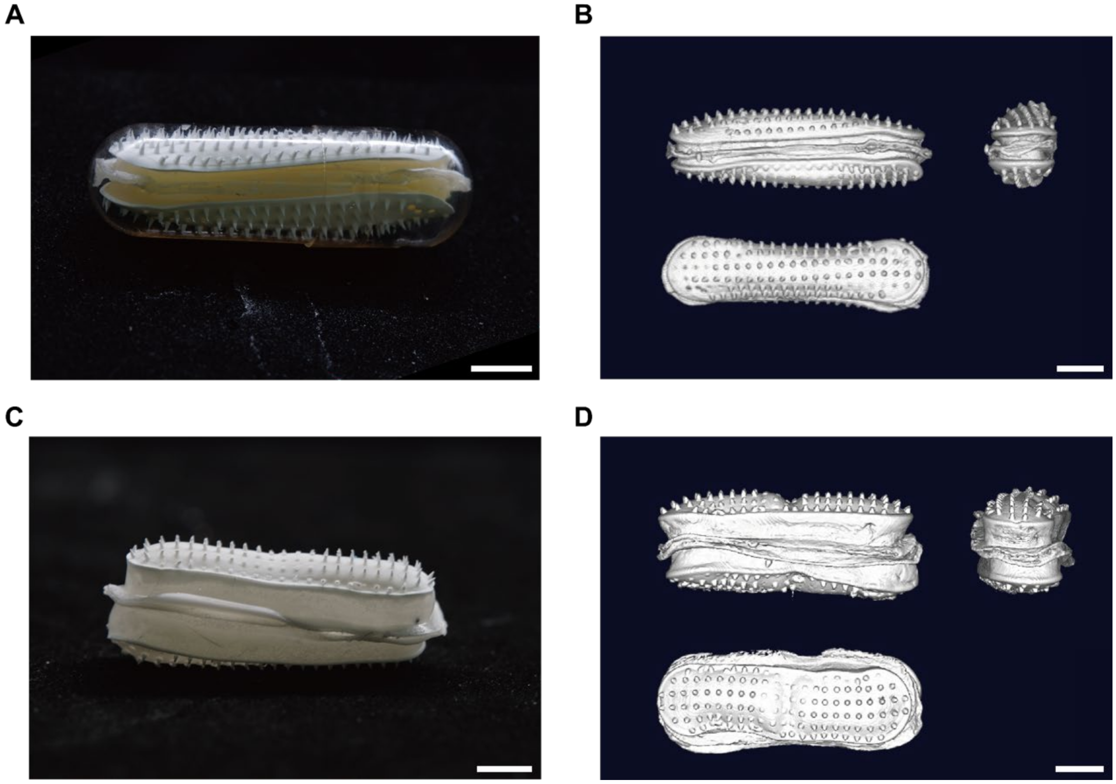

Fig. S21. Photographs and micro-CT images of X-ray contrast-loaded microneedle robots. (A) Photograph of a microneedle robot in a capsule. (B) The micro-CT three-view images of microneedle robot. (C) Photograph of the microneedle robot at the swelling equilibrium state. (D) The micro-CT three-view images of microneedle robot at the swelling equilibrium state. Scale bars, 5 mm. The images indicate that the microneedle robots are clearly visible in X-rays.

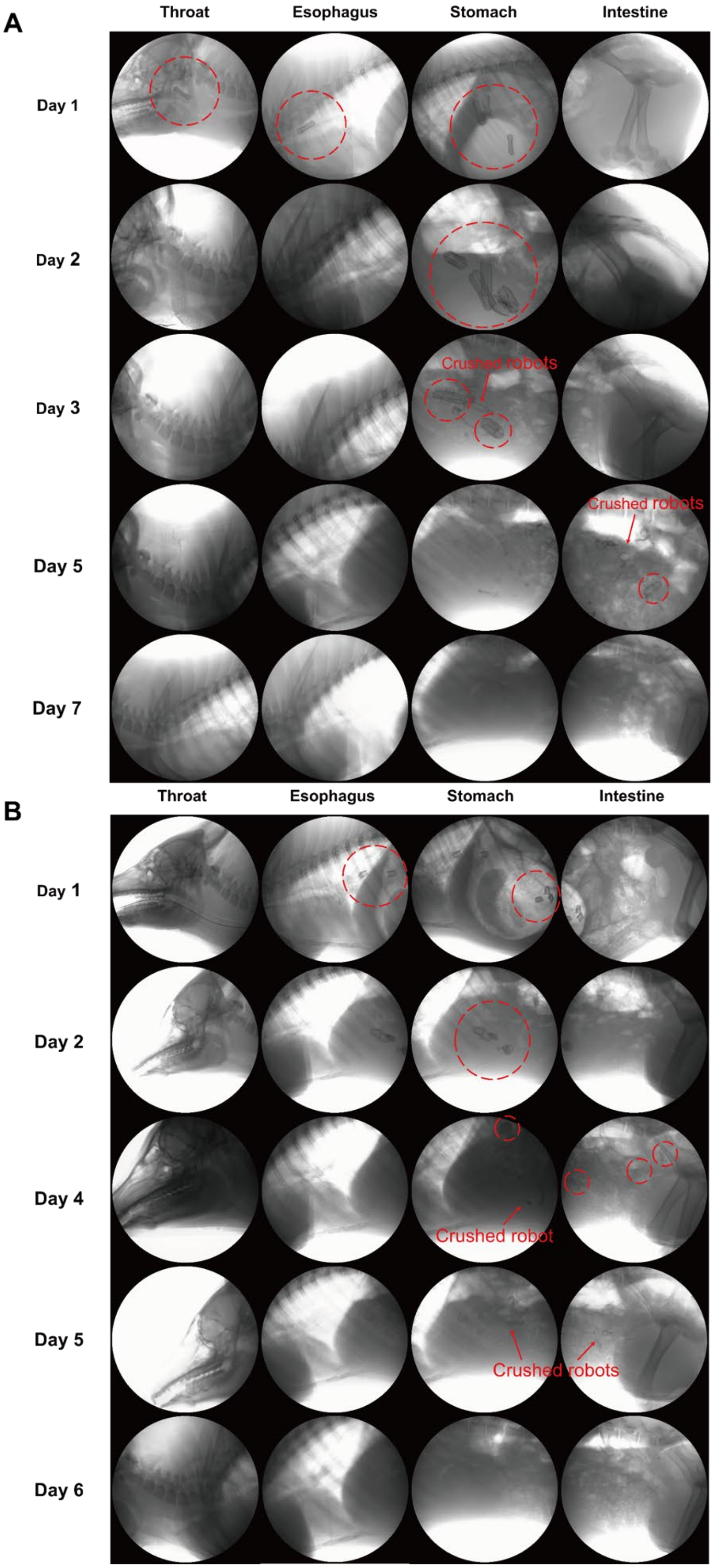

A
Throat
Esophagus
Stomach
Intestine
Day 1
Day 2
Day 3
Day 5
Day 7
Crushed robots
Crushed robots
B
Throat
Esophagus
Stomach
Intestine
Day 1
Day 2
Day 4
Day 5
Day 6
Crushed robot
Crushed robots

Fig. S22. X-ray images showing the oral delivery and excretion pathway of five 30mm-long microneedle robots (A) and five 15mm-long microneedle robots (B) in a minipig. Five microneedle robots, each measuring 30 mm in length, successfully traversed a minipig's gastrointestinal tract within a period of seven days. The X-ray images on day 1 indicated that the microneedle robots had progressed through the throat, esophagus and moved into the stomach. For the first four days, all microneedle robots were situated in the stomach, potentially due to the prolonged gastric emptying time and the narrow pylorus, which can impede the passage of commercial 00 # enteric capsules. By day 5, only one microneedle robot was observed in the intestine while the others were crushed by the chyme during the stomach contraction processes. By day 7, there were no microneedle robots or related pieces present in the X-ray images, suggesting their successful excretion from the minipig’s gastrointestinal tract. The minipig displayed no symptoms of obstruction throughout the experiment.

Subsequently, we examined the effect of the size of the microneedle robots on pyloric transit by creating five 15 mm-long microneedle robots. These shorter robots traversed the gastrointestinal tract in a reduced timeframe of six days, quicker than their 30 mm counterparts. These shorter microneedle robots remained within the stomach for only three days. On day 4, four microneedle robots were observed to have moved into the intestine and only one was crushed in the stomach. On day 5, only some pieces of the microneedle robots were left in the intestine. By day 6, no microneedle robots or related pieces presented in the X-ray images.

In summary, the shorter microneedle robots can pass through the gastrointestinal tract faster than the longer ones, possibly due to their ease of passage through the pylorus. Additionally, long gastric resident duration makes the microneedle robots more readily to be destroyed under intense stomach contractions. In this experiment, four of five 30 mm-long microneedle robots versus only one of the 15 mm-long robots were broken down in the stomach.

Nevertheless, when considering potential human translation, it is worth noting that humans typically exhibit a faster gastric emptying time and possess a more relaxed pylorus compared to minipigs. Therefore, it is plausible that microneedle robots may traverse the human gastrointestinal tract in a shorter duration and may have a reduced possibility of destruction inside the human stomach.

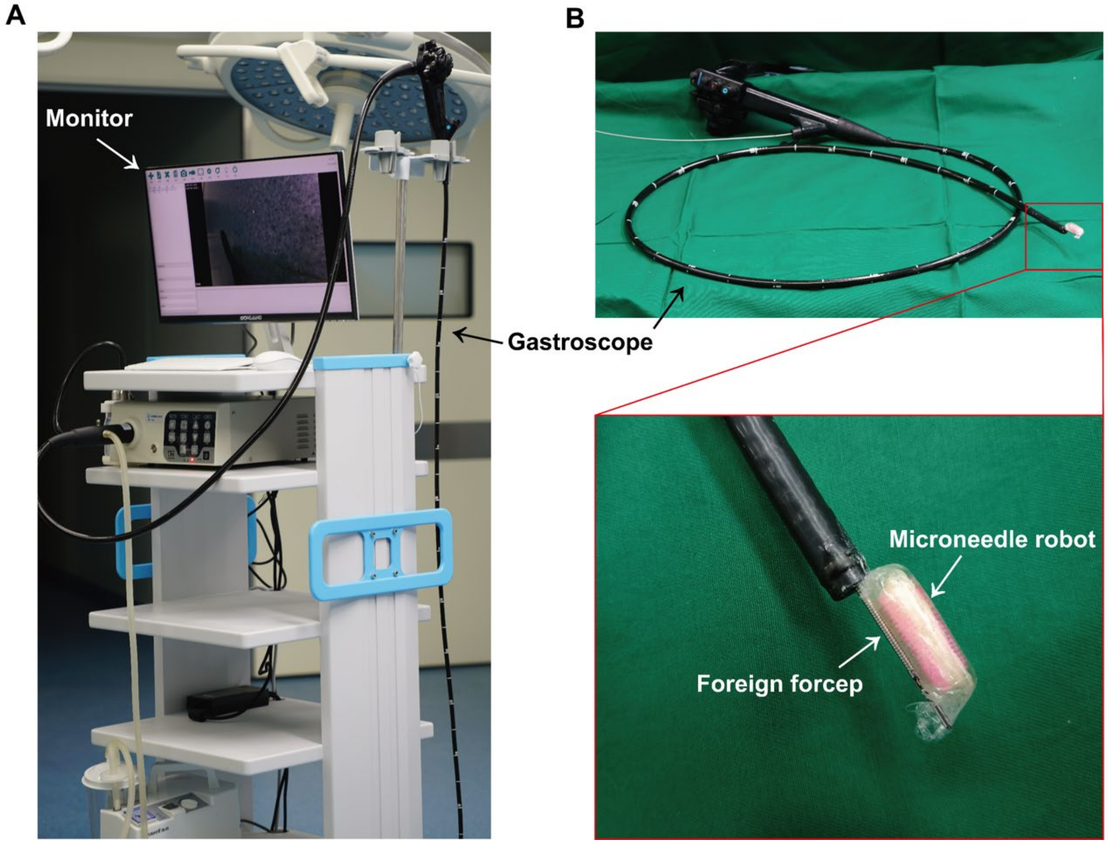

Fig. S23. Photographs of the gastroscopic delivery experiment setup.

SUPPLEMENTARY TABLES

Table S1. Gradient UPLC method for AAm and PEGDA.

Time (min)	Mobile phase A (%)	Mobile phase B (%)	Flow (mL/min)
0.00	98.0	2.0	0.400
1.00	98.0	2.0	0.400
7.00	0.0	100.0	0.400
8.00	0.0	100.0	0.400
10.00	98.0	2.0	0.400

A: water; B: acetonitrile.

Table S2. Comparison of area under curve calculations from the pharmacokinetics study *in vivo*

	Intestinal gavage (1.0 mg)	Subcutaneous injection (0.2 mg)	Robotic delivery (with barbs, 0.8 mg)	Robotic delivery (without barbs, 0.8 mg)
Mean	3241	95877	90429	15533
Standard Error	936.2	25954	27056	7979
95% Confidence Interval	1406 to 5076	45009 to 146746	37401 to 143458	0 to 31172
Number of samples	5	5	5	5

Table S3. Comparison of unpaired two-tailed t-test of 3h blood glucose changes of different delivery methods

	Robotic delivery (with barbs, 0.8 mg) vs. Intestinal gavage (1.0 mg)	Robotic delivery (with barbs, 0.8 mg) vs. Subcutaneous injection (0.2 mg)	Robotic delivery (with barbs, 0.8 mg) vs. Robotic delivery (without barbs, 0.8 mg)
P value	0.0099	0.6983	0.0226
P value summary	**	ns	ns
Number of minipigs	3	3	3

Table S4. Comparison of unpaired two-tailed t-test of normalized AUC of different delivery methods

	Robotic delivery (with barbs, 0.8 mg) vs. Intestinal gavage (1.0 mg)	Robotic delivery (with barbs, 0.8 mg) vs. Subcutaneous injection (0.2 mg)	Robotic delivery (with barbs, 0.8 mg) vs. Robotic delivery (without barbs, 0.8 mg)
P value	0.0099	0.0708	0.0226
P value summary	**	ns	*
Number of minipigs	5	5	5

Other Supplementary Material for this manuscript includes:

Movie S1. Swelling process of the microneedle robot in simulated intestinal fluids.

Movie S2. Gastroscopic delivery, swelling and intestinal peristalsis actuated mucosa penetration and retention of the microneedle robot in the porcine intestine *in vivo*.

Movie S3. Comparison of the penetration and retention performance of microneedles without barbs, with inseparable barbs and with separable barbs on *ex vivo* porcine intestine tissues.
